# Single ion mobility monitoring (SIM^2^) stitching method for high-throughput and high ion mobility resolution chiral analysis

**DOI:** 10.1007/s00216-024-05399-2

**Published:** 2024-06-27

**Authors:** Clément Chalet, Estelle Rathahao-Paris, Sandra Alves

**Affiliations:** 1 Sorbonne Université, Faculté des Sciences et de l’Ingénierie, Institut Parisien de Chimie Moléculaire (IPCM), Paris, France; 2 Université Paris-Saclay, CEA, INRAE, Médicaments et Technologies pour la Santé (MTS), Gif-sur-Yvette, France

**Keywords:** Ion mobility, Mass spectrometry, TIMS, Amino acids, Chiral analysis

## Abstract

Chiral analysis is of high interest in many fields such as chemistry, pharmaceuticals and metabolomics. Mass spectrometry and ion mobility spectrometry are useful analytical tools, although they cannot be used as stand-alone methods. Here, we propose an efficient strategy for the enantiomer characterization of amino acids (AAs) using non-covalent copper complexes. A single ion mobility monitoring (SIM^2^) method was applied on a TIMS-ToF mass spectrometer to maximize the detection and mobility separation of isomers. Almost all of the 19 pairs of proteinogenic AA enantiomers could be separated with at least one combination with the chiral references L-Phe and L-Pro. Furthermore, we extended the targeted SIM^2^ method by stitching several mobility ranges, in order to be able to analyze complex mixtures in a single acquisition while maintaining high mobility resolution. Most of the enantiomeric pairs of AAs separated with the SIM^2^ method were also detected with this approach. The SIM^2^ stitching method thus opens the way to a more comprehensive chiral analysis with TIMS-ToF instruments.

**Graphical Abstract:**

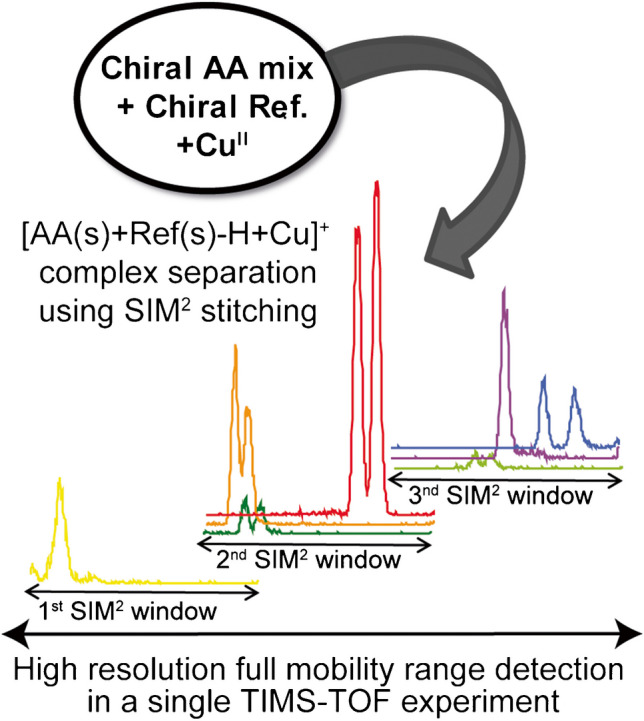

**Supplementary Information:**

The online version contains supplementary material available at 10.1007/s00216-024-05399-2.

## Introduction

Chirality poses a unique challenge in life sciences, since each individual enantiomer can have specific biological activity. Efficient chiral analysis methods are thus required. Various chiral spectroscopic (X-ray crystallography, circular dichroism, nuclear magnetic resonance) and chromatographic techniques have been successfully developed since the first experimental observations of a chiral molecule [1, 2]. Particularly, chromatography using chiral stationary phase (CSP) methods offered unrivaled enantioseparations and highly sensitive quantification (trace level determination of one enantiomer as low as 0.001 % in the presence of a very large amount of its opposite enantiomer) [3, 4].

Mass spectrometry (MS)-based approaches are also promising for chiral analysis thanks to their speed, specificity, and sensitivity, especially if hyphenated with sensitive chiral separation tools [5]. Stand-alone MS can also be a useful tool, even though it cannot directly resolve enantiomers due to its achiral environment, leading to the development of various strategies [2, 6–8]. The first step of such MS-based methods generally involves the formation of diastereomeric species, either by chemical derivatization in solution [9, 10] or by non-covalent assembly in the gas phase [8], or even a combination of both approaches [11]. Nevertheless, gas-phase complex formation is usually the simplest and least time-consuming method, as no sample preparation or compound purification is required. Although the formation of diastereomeric clusters by self-association may be a viable alternative for determining enantiomeric composition [12], one or more chiral modifiers are generally required to form diastereomeric complexes giving sufficient enantiomer differentiation [8]. Moreover, the addition of alkali or transition metal ions (e.g., Cu, Ni, or Fe) is often a way to promote rigid structures in order to increase conformational differences of diastereomer species [13–16]. Tandem mass spectrometry (MS/MS) experiments can be subsequently performed to investigate these diastereomeric complexes, allowing the distinction of enantiomers based on (even small) differences in their stereoselective fragmentation patterns [2, 6–8, 17, 18].

More recently, ion mobility coupled to mass spectrometry (IM-MS) has emerged as a promising technique to characterize (stereo)isomers [2, 6–8, 12–14, 16]. Ion mobility separates ions based on their mass-to-charge ratio (*m/z*) ratio, their size, and shape, which makes it a relevant tool for structural elucidation [19–21]. However, the separation of isomers by IM-MS remains a challenge related to the type of isomerism, often requiring high mobility resolving power [12, 22–24]. In particular, enantiomer differentiation still involves an indirect approach through the production of relatively large (and potentially weakly bound complex) diastereoisomer ions for optimal chiral recognition [11–14, 25, 26]. Trapped ion mobility spectrometry (TIMS) is the latest technology developed in the field [27]. This device has the ability to increase its ion mobility separation capacity in a targeted way by focusing on a limited mobility range. Resolving mobility power up to 200 can be achieved and baseline separation becomes possible for isomers with small differences (< 2%) in their collision cross sections (CCS, in Å^2^) [22]. The efficacy of this approach, termed single ion mobility monitoring (SIM^2^) method, has been demonstrated for the discrimination of human milk oligosaccharide isomers [24].

In this study, the potential of SIM^2^ method for chiral differentiation was explored using a TIMS-ToF mass spectrometer. Amino acids (AAs) were chosen as model compounds, given their biological relevance and abundance. Although proteinogenic amino acids are all from the L-series, D-AAs can be found in the living world: in various food products [28] such as tea [29] and human milk [30], in bacterial cells [31], as well as in agriculture [32]. Chiral characterization of AAs through derivatization [11, 25, 33–37] or formation of non-covalent complex species [13, 14, 26, 38, 39] has already been reported, but the choice of an adequate chiral selector is still a critical step, and its efficacy appears to be largely compound-dependent [14]. Chelation with various chiral selectors as cyclodextrins [13] and oligosaccharides [38] was proposed by analogy with chiral stationary phase. The use of amino acids [14, 25, 26, 40] as chiral selectors also allowed the successful characterization of chiral AAs using IM-MS. Conversely, high assembly complexes with large mobility differences are often required for chiral recognition [13, 14, 26, 40] due to the moderate resolving powers available on conventional IM-MS platforms (R∼50).

Our study focused on the characterization of non-covalent complexes of AAs with copper (Cu^II^), using L-Phe and L-Pro as chiral references, in particular on small size clusters, i.e., dimers [AA+AA-H+Cu^II^]^+^ and trimers [AA+AA+AA-H+Cu^II^]^+^, which are more likely to be produced in the gas phase. Previous studies [14, 26] have described aromatic amino acids such as Phe as good choices as chiral references, because interactions between the aromatic side chain and the chiral analyte could contribute to chiral recognition [8]. Additionally, cyclic amino acids should induce conformational rigidity; for example using Pro as a chiral selector has been shown to provide enantioselectivity [40]. Here, the combination of both L-Phe and L-Pro was chosen as chiral references to investigate the chiral separation of a large set of AAs by IM-MS. Chiral differentiation was first assessed by analyzing each enantiomeric pair of AAs under optimal ion mobility separation conditions with the SIM^2^ method [24]. Then, an innovative high-throughput and high mobility resolution method was developed to extend the mobility range without losing mobility resolution, allowing simultaneous analysis of multiple pairs of enantiomeric AAs with different mobilities. This approach, named SIM^2^ stitching, should provide in a single acquisition the highest ion mobility separation available with high MS sensitivity.

## Experimental section

### Chemicals

All chiral L- and D-amino acids (alanine (Ala), arginine (Arg), asparagine (Asn), aspartic acid (Asp), cysteine (Cys), glutamine (Gln), glutamic acid (Glu), histidine (His), isoleucine (Ile), leucine (Leu), lysine (Lys), methionine (Met), phenylalanine (Phe), proline (Pro), serine (Ser), threonine (Thr), tryptophane (Trp), tyrosine (Tyr), valine (Val)), and copper(II) chloride (CuCl_2_) were purchased from Sigma-Aldrich (Merck KGaA, Darmstadt, Germany). Ultrapure water was produced with a Select HP water purification system (Purite France Eau, Lormont, France). Methanol (CH_3_OH) was obtained from VWR Chemicals (Fontenay-sous-Bois, France). The ESI-L low concentration tuning mix (G1969-85000) for TIMS-ToF calibration was purchased from Agilent Technologies (Santa Clara, CA, USA).

Stock solutions of chiral amino acids were prepared in H_2_O at a concentration of 10 mM (except Tyr at 1 mM) and stored at -20 °C. Working solutions of 1 mM in H_2_O were used for sample preparation and kept at +4 °C. Except when otherwise stated, AAs and CuCl_2_ were diluted in H_2_O/CH_3_OH (1:1, v/v) to obtain a final concentration of 5 µM each and directly infused into the mass spectrometer (3 µL/min flow rate).

### Ion mobility and mass spectrometry

All IM-MS experiments were conducted on a TIMS-ToF^TM^ mass spectrometer (Bruker Daltonics, Bremen, Germany) using electrospray ionization (ESI) in positive mode. Nitrogen was used as both spray and drift gas. All data were acquired in the m/z range of 100 to 1600 Da.

Source parameters were set as follows: capillary voltage, -4500 V; end plate offset, 500 V; dry gas flow, 3 L/min; nebulizer gas pressure, 3 psi; capillary temperature, 275 °C. The following MS parameters were used: funnel 1 RF, 250 V_pp_; funnel 2 RF, 350 V_pp_; isCID energy, 0 V; multipole RF, 800 Vpp; deflection delta, 100 V; ion energy, 5 eV; collision energy, 5 eV; collision RF, 1100 Vpp; transfer time, 65 µs; pre pulse storage, 10 µs. TIMS parameters were set as follows: collision cell in, 200 V; Δ1, -60 V; Δ2, −140 V; Δ3, 140V; Δ4, 70 V; Δ5, 0 V; Δ6, 100 V; ion charge control (ICC) was set to 0.10×10^6^, and accumulation time was locked to the mobility range. All MS and IMS parameters were controlled through the oTof control 6.2 software (Bruker Daltonics).

Daily external calibrations in *m/z* (quadratic mode) and in reduced mobility values (linear mode) were carried out using the ESI-L low concentration tuning mix solution. CCS values were then calculated from the measured reduced mobilities.

The separation capacity of the TIMS device was assessed based on the ΔCCS% value, defined as the CCS difference between two peaks A and B divided by the average of their CCS:

$$\Delta \text{CCS}\%\;=\;2\left(\frac{{\text{CCS}}_{\text{B}}\;-\;{\text{CCS}}_{\text{A}}}{{\text{CCS}}_{\text{B}}\;+\;{\text{CCS}}_{\text{A}}}\right)\times 100$$

Alternatively, the resolution (*R*) can also be determined from the measured mobilograms (1/*K*_0_ vs intensity), according to the equation *R* = *K*_0_/Δ*K*_0_, where *K*_0_ is the reduced mobility of a peak and Δ*K*_0_ its peak width at half height (or full width at half maximum, FWHM). All data were processed with DataAnalysis v. 5.3 (Bruker Daltonics).

TIMS separation involves consecutive events of accumulation and elution, and adjusting their parameters can increase the separation capacity of the mobility cell [27]. Briefly, during mobility separation, ions are pushed through the cell by the buffer gas and trapped by an axial electric field gradient, which stops them at an equilibrium position. The electric field is then gradually decreased by lowering the exit potential (*V*_out_), and the ions are sequentially liberated into the time-of-flight mass analyzer. The exit voltage range and the speed at which this voltage is decreased (ramp time) define the slope of the electric field gradient, which affects the resolving power of the TIMS.

Thus, different mobility modes are available (moderate, high, and maximum resolution, together termed as Imex Technology), each corresponding to fixed spectrum rate values (Hz). The mobility range 1/K_0_ (V.s.cm^−2^) is chosen by the user, and the ramp time (ms) is adjusted to match the spectrum rate. By applying the maximum resolution conditions, the SIM^2^ method uses narrow ion mobility range detection centered on a targeted ion, with a small mobility window of 0.10 V.s.cm^−2^ (scan rate around 3 Hz), which allows enhanced ion mobility resolution and sensitivity, albeit with a longer analysis time.

In the present study, we also used an alternative approach to SIM^2^, coined as SIM^2^ stitching, which consists in the continuous collection of multiple adjacent SIM^2^ windows with variable mobility and overlapping ranges. SIM^2^ stitching thus allows the coverage a wide mobility range while maintaining at the same time high-resolution conditions in a single acquisition (see Electronic Supplementary Material Scheme S1).

## Results and discussion

### SIM^2^ mode to evaluate chiral differentiation of AA enantiomers

The SIM^2^ method (i.e., using a narrow ion mobility range) was applied in order to detect each non-covalent complex ion formed from AA enantiomers under high mobility resolution conditions. Each enantiomeric pair of AAs was analyzed in the presence of two chiral selectors L-Phe and L-Pro and Cu^II^ in two steps: first alone with the two selectors, then as an equimolar mixture of both enantiomers. Under positive electrospray ionization, copper adduct [M-H+Cu^II^]^+^ complexes were detected, including dimers and trimers, with M for each possible combination of the analyte and chiral references: AA/Pro, AA/Phe and AA/AA for dimers, and AA/Pro/Pro, AA/Phe/Pro, AA/Phe/Phe, AA/AA/Pro, AA/AA/Phe, and AA/AA/AA for trimers. One exception is Cys, for which covalent dimers of cystine (Cys,Cys-2H) were found. In our conditions, larger copper adduct complexes such as binuclear copper-bound tetrameric ions as reported by Yu and Yao (with Trp as chiral selector) were not detected [14]. Sodiated and/or potassied species could also be detected for some AAs, but copper adducts showed the most effective chiral distinction. Negative ion polarity was also tested but the formation of AA complexes as well as their chiral differentiation was less efficient compared to positive ion mode (see Electronic Supplementary Material Fig. S1).

**Fig. 1:**
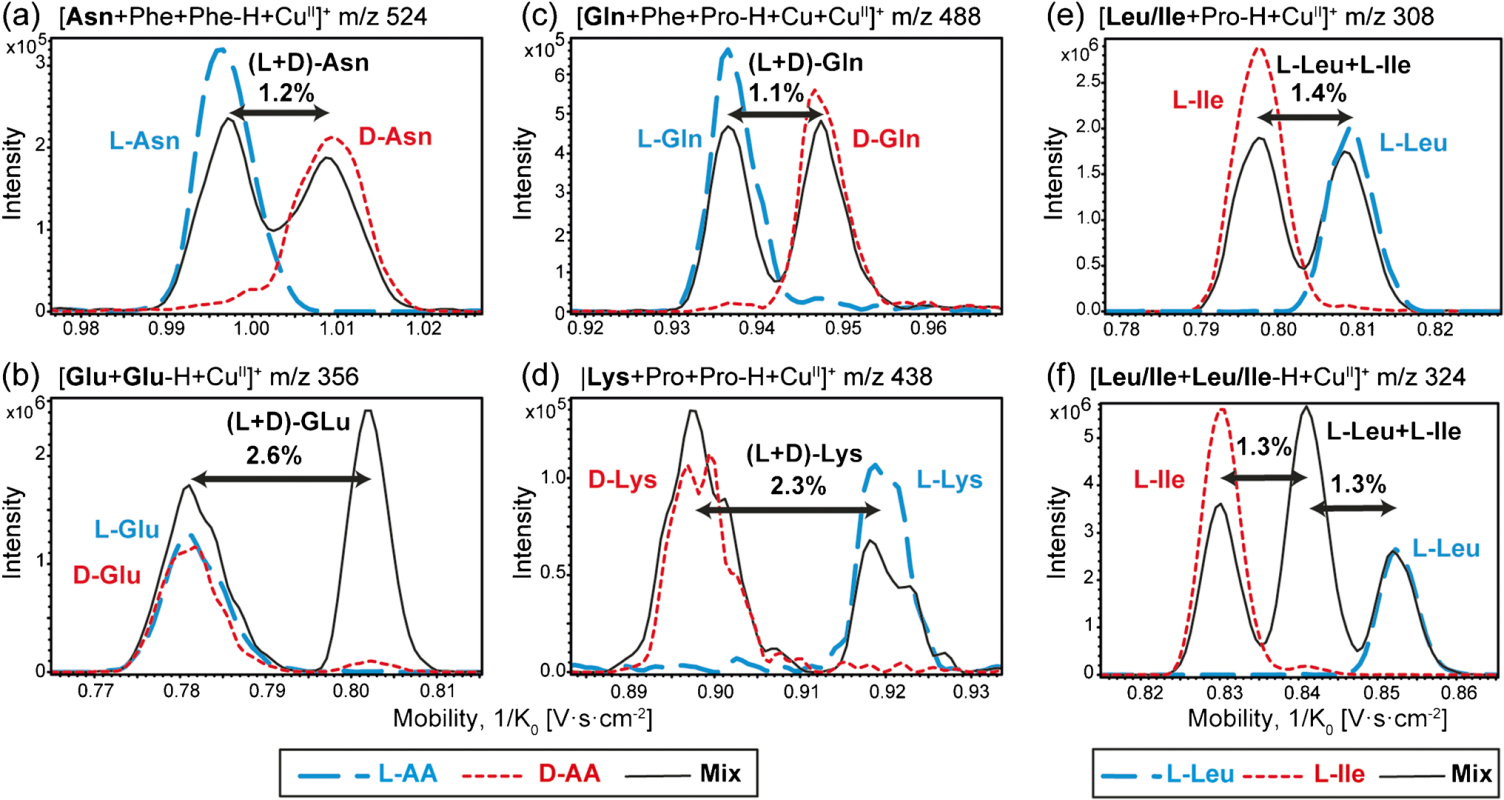
Extracted ion mobility spectra from IM-MS analyses of copper complexes of **a** asparagine (Asn), **b** glutamic acid (Glu), **c** glutamine (Gln), and **d** lysine (Lys) enantiomers, and the L-enantiomer complexes of leucine (Leu) and isoleucine (Ile) isomers, **e** [Leu/Ile+Pro-H+Cu^II^]^+^ and **f** [Leu/Ile+Leu/Ile-H+Cu^II^]^+^. Signals obtained from three different acquisitions using SIM^2^ mode are shown in each figure: L-AA and D-AA alone and an equimolar mixture of L- and D-AA in (**a**, **b**, **c**, and **d**) and L-Leu, L-Ile alone, and the equimolar mix of both isomers (**e** and **f**) with the presence of L-Pro, L-Phe, and Cu^II^. ΔCCS% values obtained from the analysis of the equimolar mixture solutions are indicated in each graph

The ability of IM-MS to separate AA enantiomers was evaluated based on the ΔCCS% value, corresponding to the difference in CCS (in %) between two diastereomeric complex ions. As previously reported, SIM^2^ mode with a TIMS device allows baseline separation for two mobility peaks with ΔCCS% >1.0% [24]. ΔCCS% values between 0.5 and 1.0% usually characterize only partial separations, depending on mobility peak shape and intensity. Results obtained from SIM^2^ analyses of each enantiomeric pair of AAs are summarized in Table 1, displaying the ΔCCS% values for the various diastereomeric dimer and trimer ions detected (full results are listed in Electronic Supplementary Material Table S1). Examples of ion mobility separation of L- from D- enantiomers for some AAs are also shown in Fig. 1 and the Electronic Supplementary Material Fig. S2.

**Table 1:**
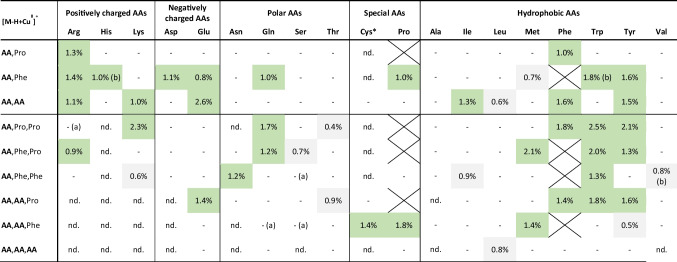
ΔCCS% values calculated from CCS values of non-covalent complexes formed with L- and D-amino acids with Cu^II^ and L-Phe & L‑Pro as chiral references. Good mobility separations are highlighted in green and partial separations in grey. (CCS values can be found in Table S1 in SI.)

(nd)  Cluster ions are not detected

(-) Ions are detected but there is no ion mobility separation or ΔCCS% values are too low for proper separation (typically ΔCCS%  < 0.5%)

(a) No (poor) mobility separation due to additional peaks when analyzing the enantiomer mix compared to single enantiomer analysis

(b) Multiple signals are detected for one compound

*The ions detected for Cys could correspond to its reduced dimer, i.e., [(Cys+Cys-2H)+ref(s)-H+Cu^II^]^+^

ΔCCS% values greater than 1% were obtained for at least one complex ion for almost all AAs, indicating that baseline mobility separation was achieved for these enantiomeric AA pairs. Exceptions are observed for Thr, Ser, Leu, and Val enantiomers, for which the highest ΔCCS% values were less than 1% (i.e., 0.7% ≤ ΔCCS% ≤ 0.9%). Therefore, only partially resolved mobility signals were obtained for these latter enantiomeric pairs of AAs. Unsurprisingly, no mobility separation was obtained for Ala, the smallest AA.

Self-association, i.e., a complex formed with at least one pair of AA enantiomers, can also be used for efficient chiral recognition [12]. An illustrative example for Glu is shown in Fig. 1b with [Glu+Glu-H+Cu^II^]^+^. When analyzing the Glu enantiomeric mixture, ion mobility displays two peaks. The first one corresponds to the mixture of both homochiral L-Glu/L-Glu and D-Glu/D-Glu dimers, which are enantiomers of each other and whose mobility peaks thus overlap. A second signal, distinct from the first one, corresponds to the heterochiral complex L-Glu/D-Glu, which makes it possible to demonstrate the presence of both enantiomers.

Interestingly, self-association also allowed the distinction of two isomers, leucine and isoleucine. The characterization of these isomers represents a constant challenge in the analysis of AAs. Recently, the formation of non-covalent trinuclear copper complexes has been proposed to enhance the differentiation of enantiomers, including those of Leu and Ile [26]. In our work, we were able to separate Leu and Ile isomers of the same chirality with two combinations of complexes, namely with L-Pro as chiral selector and as a self-association dimer (Fig. 1e and f, respectively). The enantiomeric separation of Leu or Ile appeared more complicated, since only the dimer [Ile+Ile-H+Cu^II^]^+^ gave a sufficient enantiomer mobility separation (ΔCCS% of 1.3%, Table 1). When analyzing the mixture of the enantiomers of both Leu and Ile isomers, distinction between the four enantiomers/isomers molecules was challenging. The most promising combination was the formation of the self-associated dimers [AA+AA-H+Cu^II^]^+^, but even then, their ΔCCS% values were below 1% (see Table 1, Fig. S3 and Table S2 in Electronic Supplementary Material). Nevertheless, the homochiral dimer signals could be used to characterize the presence of Leu and/or Ile isomers depending on the measured CCS values. The distinction between the L and D enantiomers of the two AAs was not possible, but the last heterochiral dimer signal, although poorly resolved, could reflect the presence of both L–Leu and D-Leu enantiomers.

Overall, there is no clear trend about optimal complex ion formation in terms of preferential chiral reference (L-Phe *vs.* L-Pro) or copper-bound cluster size (dimer *vs.* trimer ions, self-association or chelation with chiral selector(s)). Optimal separation conditions appear to be highly compound-dependent, which can limit the general application of this approach for chiral analysis. It is therefore important to assess different chiral selectors (or the combination of several of them) to find a suitable combination for the efficient separation of a specific set of analytes. In contrast to recent results [35], no particular mobility order of the D and L enantiomers can be concluded from our results, and no dependence on AA structure or size was observed (see the examples of Glu-containing species in Electronic Supplementary Material Fig. S2, for which the elution order differs depending on the clusters). Nevertheless, efficient ion mobility separation was achieved for almost all AA cluster ions, only short-chain aliphatic AAs were not readily separated (Table 1 and Fig. S2 in Electronic Supplementary Material). Higher assembly complexes (e.g., using cyclodextrins as chiral selectors and/or larger clusters as tetramers or larger [13, 14, 26]) or derivatization are likely required for efficient chiral analysis of these low-mass AAs (e.g., for Ala [25, 35, 37]). Finally, measurable differences in mobility across the almost AA enantiomers suggest that quantitative determination of enantiomeric excess can be achieved for almost all chiral AAs using high-resolution ion mobility separation. The main limitation could arise from the differences in MS response between enantiomers in copper complexes, which could complicate the analysis of complex mixtures and the relative quantification of chiral molecules (see, for instance, the variation of relative intensity of [Asn+Phe+Phe-H+Cu^II^]^+^ ions between D and L form in Fig. 1a).

### SIM^2^ stitching to cover a large ion mobility range

Further investigations were made on chiral AA mixtures using SIM^2^ stitching acquisition. Here, the SIM^2^ method uses a narrow ion mobility range centered on the CCS value of the species of interest, which provides a high mobility resolving power (up to 200) [24]. This method is thus limited to targeted analyses (see Experimental section and Scheme S1 in Electronic Supplementary Material). Consequently, high mobility resolution characterization of multiple compounds in a single acquisition is not as straightforward using a TIMS-ToF mass spectrometer. Several compromises have been proposed, such as using moderate mobility resolution for a wide range detection in a single acquisition from large size diastereomeric species [35] or performing serial acquisitions at different mobility ranges under high-resolution conditions to cover almost all chiral AA mobilities [34]. Here, we introduce the SIM^2^ stitching method, which aims to cover a wide mobility range while maintaining high mobility resolution and sensitivity of TIMS device. This method would allow the exhaustive detection of many species having different mobilities in a single acquisition. This approach is analogous to single ion monitoring, or spectral stitching, using ICR cell detection, through the collection of multiple adjacent and overlapping SIM^2^ windows [41, 42].

The optimization of the experimental conditions favoring the formation of copper adducts was first carried out before assessing SIM^2^ stitching for the chiral analysis of AAs. Different concentrations of both chiral selectors (L-Phe and L-Pro) and Cu^II^ (from 1 to 10 µM) were tested in the analysis of a mixture of five chiral AAs (Arg, Gln, Met, Trp, and Tyr, each enantiomer at 1 µM). An excess of chiral references was found to favor the formation of complexes under ESI conditions (see Fig. S4 in Electronic Supplementary Material). The width of the SIM^2^ windows as well as the overlap between two adjacent windows are important features to consider for SIM^2^ stitching analysis in order to ensure good detection coverage of all the analytes present in a given sample. Different window widths from 0.1 to 0.2 V.s.cm^−2^ have been tested with two enantiomer mixtures of AAs (Arg, Asp, Cys, Gln, Ile, Ser, Trp, and Val in Fig. S5 and Asn, Glu, His, Leu, Lys, Met, Thr, and Tyr mixture in Fig. S6 of Electronic Supplementary Material). The width of the mobility window has no impact on the mobility resolution, which is expected given that the scan rate is kept constant by the TIMS instrument (see “Experimental section” section). However, a greater sensitivity is observed by decreasing the width of the SIM^2^ window, which is especially crucial for detecting low-intensity peaks of non-covalent complexes. This gain of sensitivity is likely due to an increase in the signal-to-noise ratio by minimizing the space charge in the TIMS cell.

**Fig. 2:**
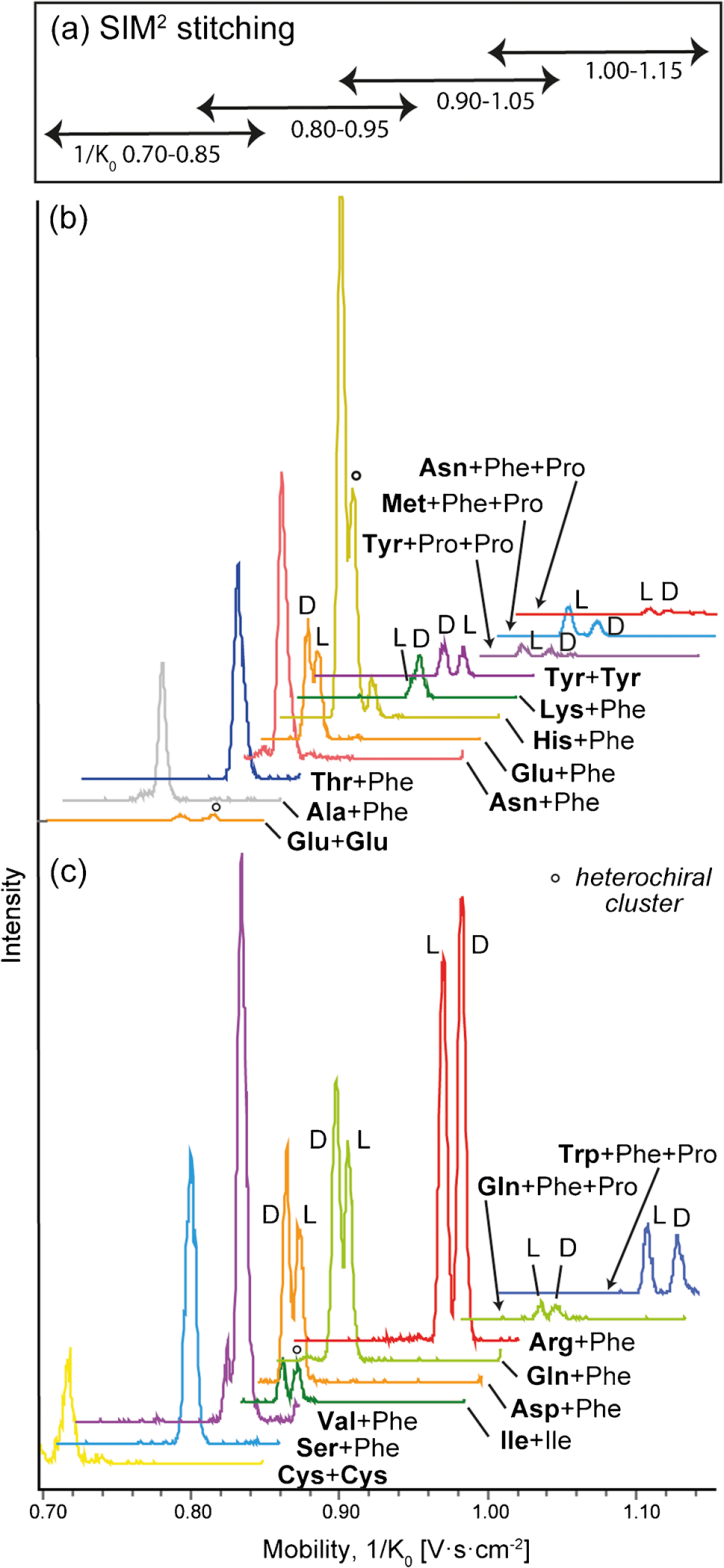
**a** Different ion mobility ranges (with a 1/*K*_0_ window of 0.15 V.s.cm^−2^, overlapped by 0.05 V.S.cm^−2^) used in SIM^2^ stitching analysis. Selected extracted ion mobility spectra (stacked view) from SIM^2^ stitching analyses of two mixes of AA enantiomers: **b** Arg, Asp, Cys, Gln, Ile, Ser, Trp, and Val; **c** Ala, Asn, Glu, His, Leu, Lys, Met, Thr, and Tyr. Each AA (L and D enantiomers) was at a concentration of 1 µM, L-Phe, and L-Pro were at 10 µM and Cu^II^ at 20 µM. Full results are shown in Electronic Supplementary Material Table S3 and S4 and Fig. S6 to S9

The following SIM^2^ stitching conditions were finally chosen in order to obtain optimized detection in terms of mobility resolution and sensitivity: mobility windows with 1/K_0_ width of 0.15 V.s.cm^−2^ and an overlap of 0.05 V.s.cm^−2^. Data acquisition was done using four adjacent segments and 60 s of acquisition duration for each sample, over a mobility range from 0.70 to 1.15 V.s.cm^−2^ (i.e., 0.70–0.85, 0.80–0.95, 0.90–1.05, 1.00–1.15 V.s.cm^−2^). The analyses were then performed on two distinct mixes of enantiomeric pairs of AAs, and selected extracted ion mobility spectra are reported for all pairs of AAs in Fig. 2

Data processing is an important step to extract information of interest from SIM^2^ stitching acquisition. Extracted ion mobility signals can be obtained either by analyzing separately each mobility segment or by simultaneously processing the entire acquisition over all ion mobility ranges. The results shown in Fig. 2 (as well as Fig. S6 and S8 in Electronic Supplementary Material) were obtained using the first method, while Fig. S7 and S9 in Electronic Supplementary Material show the results obtained by processing the entire acquisition. Signals extracted with the latter method display better peak shapes, with most of the peaks displaying higher intensities. This increase in sensitivity can be explained by mobility signals falling within an overlap, i.e., two adjacent mobility ranges, which induce higher signal intensity. However, processing the entire analysis creates artifact signals at the end of each SIM^2^ segment (i.e., at 0.85, 0.95 and 1.05 V.s.cm^−2^), which may interfere with peaks of interest (see, for example, the Arg/Pro dimer in Fig. S8, Electronic Supplementary Material). The cause of these “edge effects” artifacts is unclear and could be due to changes of potential in the TIMS cell, which would cause the baseline rise at the end of the mobility detection range.

Overall, a total of 91 complexes were detected out of 153 possible combinations (59%) during SIM^2^ stitching analysis of the mixes of AAs (43 complexes from the mix of Arg, Asp, Cys, Gln, Ile, Ser, Trp, and Val and 48 from the mix of Ala, Asn, Glu, His, Leu, Lys, Met, Thr, and Tyr; see Table S3 and Table S4 in Electronic Supplementary Material). Out of those 91 complexes, 21 were sufficiently separated. There is also a 27% difference in the detection of complexes using SIM^2^ stitching compared to SIM^2^ analysis of individual solutions of enantiomers (91 *vs.* 125 complexes detected). However, this difference is quite limited not directly related to the method used but rather to the composition of the complex mixture analyzed with SIM^2^ stitching, in particular the associated competition for ionization and the formation of non-covalent complexes. Under these conditions, complexes containing two or more molecules of AAs are less likely to be detected compared to individual solutions, since all AAs present in solution can potentially interact with each other to form copper complexes. Moreover, possible isobaric interferences in such mixtures may also occur, which cannot always be resolved by the TOF analyzer (here, ion mobility spectra were extracted at a given *m/z* value with an error of ±0.01 Da). These limitations should nonetheless be compensated using the SIM^2^ stitching, which minimizes space charge effects, thus providing increased sensitivity.

## Conclusion

High-resolution ion mobility spectrometry is essential for the separation of structurally related compounds, in particular for the characterization of chiral compounds. The SIM^2^ method takes fully advantage of the high mobility resolution capabilities of the TIMS-ToF mass spectrometer. In this work, this approach allowed the chiral characterization of almost all the amino acids studied through the formation of non-covalent copper complexes with L-Phe and/or L-Pro as chiral references.

Nevertheless, a drawback of the SIM^2^ method is that its maximum resolution and intensity are obtained with narrow mobility detection windows, limiting its use to targeted analyses. Here, we proposed a high-throughput and high-resolution ion mobility strategy by stitching multiple adjacent ion mobility ranges, which allows the comprehensive analysis of a mixture of AAs during a single acquisition. In our study, this SIM^2^ stitching approach allowed the simultaneous characterization of almost all enantiomeric pairs of AAs.

Overall, SIM^2^ stitching emerges as a promising method for the rapid and comprehensive characterization of any mixture of compounds, in particular if coupled with automated sample injection techniques such as flow injection analysis (FIA) using the autosampler of a liquid chromatography system.

### Supplementary Information

Below is the link to the electronic supplementary material.

Supplementary file1 (PDF 2701 KB)
