# Supplementary material for: Single ion mobility monitoring (SIM2) stitching method for high-throughput and high ion mobility resolution chiral analysis
Source: Anal Bioanal Chem. 2024 Jun 27;416(20):4581–9. doi: 10.1007/s00216-024-05399-2 (PMC11294385; doi:10.1007/s00216-024-05399-2)

Supplementary information

Single Ion Mobility Monitoring (SIM^2) stitching method for high throughput and high ion mobility resolution chiral analysis

Clément Chalet[1], Estelle Rathahao-Paris[1,2]*, Sandra Alves[1,*]

[1] Sorbonne Université, Faculté des Sciences et de l'Ingénierie, Institut Parisien de Chimie Moléculaire (IPCM), 75005 Paris, France

[2] Université Paris-Saclay, CEA, INRAE, Département Médicaments et Technologies pour la Santé (DMTS), 91191 Gif-sur-Yvette, France

* Corresponding authors: sandra.alves@sorbonne-universite.fr and estelle.paris@inrae.fr

ORCID numbers
Clément Chalet: 0000-0001-7638-8564
Estelle Rathahao-Paris: 0000-0002-7271-7372
Sandra Alves: 0000-0003-0063-8760

SUMMARY OF CONTENTS

Scheme 1. Schematic description of differents tested IM-MS acqusitions.

Fig. S1. Preliminary results from chiral analysis of the ion mobility separations studied in negative and positive ion mode for Phe, Pro and PhG enantiomers. Note that Phenylglycine is a non proteoigenic amino acid used in a previous study, tested as a chiral reference alongside Phe and Pro.

Fig. S2. Examples of the ion mobility separations for all chiral amino acids studied either as dimer and trimer ions. Individual enantiomer solutions (blue and red lines for L and D enantiomer, respectively) are at a concentration of 5 µM whereas equimolar enantiomer mixes (purple line) were at 2.5 µM each (with L-Phe, L-Pro chiral selectors and Cu^{II} always fixed at 5 µM). ΔCCS values (in %) were obtained by calculating the difference between CCS of two isomer peaks in mixture. IM-MS analyses were performed using SIM^2 analysis (see experimental section).

Table S1. CCS (in Å^2) and ΔCCS% values of amino acid enantiomers (2.5 µM each) with L-Phe, L-Pro and Cu^{II} (at 5 µM concentration). Good separations are highlighted in green, partial separations are highlighted in orange and insufficient or absent separations are highlighted in grey. Purple font indicates self-association, i.e. the ion mobility signals do not correspond to L- or D-enantiomer but to heterochiral versus homochiral chiral cluster ions.

Excluding Phe and Pro amino acids (as their L-enantiomers are used as chiral references), among 153 possible complexes, 125 are detected (82%), and 30 of them are separated by ion mobility experiments under SIM^2 mode (24% of detected complexes, 20% of all possible complexes), and 10 are partially resolved.

Fig. S3. Extracted ion mobility signals at *m/z* 324 ([AA,AA-H+Cu^{II}]$^+$) of various mixes of L and/or D-Ile and Leu isomers. IM-MS analyses were performed using SIM^2 analysis (see experimental section).

Table S2. Ion mobility separations obtained for L and D Leu and Ile isomers using SIM^2 analysis of equimolar isomer mixes, expressed as ΔCCS% values.

Fig. S4. Multiple SIM^2 analysis (mobility window width of 0.10 V.s/cm^2) of a mix of L- and D-Arg, Gln, Met, Trp and Tyr (each enantiomer at 1 µM) with increasing amounts of Cu^{II}, L-Phe and L-Pro (left, 1 to 10 µM) and increasing amounts of L-Phe and L-Pro with 10 µM of Cu^{II} (right, 1 to 10 µM).

Fig. S5. Multiple SIM^2 analysis for the mobility separation of an equimolar mixture of Arg, Asp, Cys, Gln, Ile, Ser, Trp and Val enantiomers with fixed reduced mobility ($1/K_0$) window widths of 0.10 V.s.cm^{-2} (i.e. ranges of 0.70-0.80, 0.76-0.86, 0.82-0.92, 0.88-0.98, 0.94-1.04, 1.00-1.10, 1.06-1.16 ranges, lighter), widths of 0.15 V.s.cm^{-2} (0.70-0.85, 0.80-0.95, 0.90-1.05, 1.00-1.15 ranges) and widths of 0.20 V.s.cm^{-2} (0.70-0.90, 0.85-1.05, 1.00-1.20 ranges, darker) see **Scheme 1**. Each AA enantiomer was at a concentration of 1 µM, and L-Phe, L-Pro chiral references and Cu^{II} at 20 µM.

Fig. S6. Multiple SIM^2 analysis of an equimolar mix of Asn, Glu, His, Leu, Lys, Met, Thr and Tyr enantiomers using fixed mobility window widths of 0.10 V.s.cm^{-2} (i.e. ranges of 0.70-0.80, 0.76-0.86, 0.82-0.92, 0.88-0.98, 0.94-1.04, 1.00-1.10, 1.06-1.16 V.s.cm^{-2}, lighter), 0.15 V.s.cm^{-2} (0.70-0.85, 0.80-0.95, 0.90-1.05, 1.00-1.15 V.s.cm^{-2}) and 0.20 V.s.cm^{-2} (0.70-0.90, 0.85-1.05, 1.00-1.20 V.s/cm^2, darker) see **Scheme 1**. Each AA enantiomer was at a concentration of 1 µM, L-Phe, L-Pro chiral references and Cu^{II} at 20 µM.

Table S3. Results from the SIM^2 stitching analysis of an enantiomeric mix of Arg, Asp, Cys, Gln, Ile, Ser, Trp and Val (1 µM each), L-Phe and L-Pro (10 µM each) and Cu^{II} (20 µM).

Table S4. Results from the SIM^2 stitching analysis of an enantiomeric mix of Ala, Asn, Glu, His, Leu, Lys, Met, Thr and Tyr (1 µM each), L-Phe and L-Pro (10 µM each) and Cu^{II} (20 µM).

Fig. S7. Extracted ion mobility spectra of the complex ions detected from the SIM^2 stitching analysis of an enantiomeric mix of Arg, Asp, Cys, Gln, Ile, Ser, Trp and Val (1 µM each), L-Phe and L-Pro (10 µM each) and Cu^{II} (20 µM). Note that the reported ion mobility signals are extracted only from a mobility segment that include each ion of interest (either 0.70-0.85, 0.80-0.95, 0.90-1.05 or 1.00-1.15 V.s/cm^2, **Scheme S1**) in contrast to **Fig. S8** where the same ion mobility spectra at given *m/z* ratio are averaged onto the entire acquisition (including the four ion mobility range segments).

Fig. S8. Extracted ion mobility spectra of the ions detected from the SIM^2 stitching analysis of an enantiomeric mix of Arg, Asp, Cys, Gln, Ile, Ser, Trp and Val (1 µM each), L-Phe and L-Pro (10 µM each) and Cu^{II} (20 µM) (4 mobility segments: 0.70-0.85, 0.80-0.95, 0.90-1.05 and 1.00-1.15 V.s/cm^2, **Scheme S1**). The ion mobility signals are extracted from the entire acquisition. Note that artifacts signals ("edge effects") appear using this data processing (see **Fig. S7** *versus* **Fig. S8**), and are highlighted at $1/K_0$ values of 0.85, 0.95 and 1.05 V.s/cm^2 (limits of each SIM^2 window range). (Inserted) Zoom on the extracted ion mobility signals for the complex [Arg,Pro-H+Cu^{II}]$^+$ showing the mobility shift induced by the artifact signal processing (i.e. extracted from the entire acquisition).

Fig. S9. Extracted ion mobility spectra of the ions detected from the SIM^2 stitching analysis of an enantiomeric mix of Ala, Asn, Glu, His, Leu, Lys, Met, Thr and Tyr (1 µM each), L-Phe and L-Pro (10 µM each) and Cu^{II} (20 µM). Note that the reported ion mobility signals are extracted only from a mobility segment that include each ion of interest (either 0.70-0.85, 0.80-0.95, 0.90-1.05 or 1.00-1.15 V.s/cm^2, **Scheme S1**) in contrast to **Fig. S10** where the ion mobility spectra at given *m/z* ratio are averaged onto the entire acquisition.

Fig. S10. Extracted ion mobility spectra of the ions detected from the SIM^2 stitching analysis of an enantiomeric mix of Ala, Asn, Glu, His, Leu, Lys, Met, Thr and Tyr (1 µM each), L-Phe and L-Pro (10 µM each) and Cu^{II} (20 µM) (4 mobility segments: 0.70-0.85, 0.80-0.95, 0.90-1.05 and 1.00-1.15 V.s/cm^2, **Scheme S1**). The ion mobility signals are extracted from the entire acquisition. Note that artifacts signals ("edge effects") appear using this data processing (see **Fig. S9** *versus* **Fig. S10**), and are highlighted at $1/K_0$ values of 0.85, 0.95 and 1.05 V.s/cm^2 (limits of each SIM^2 window range).

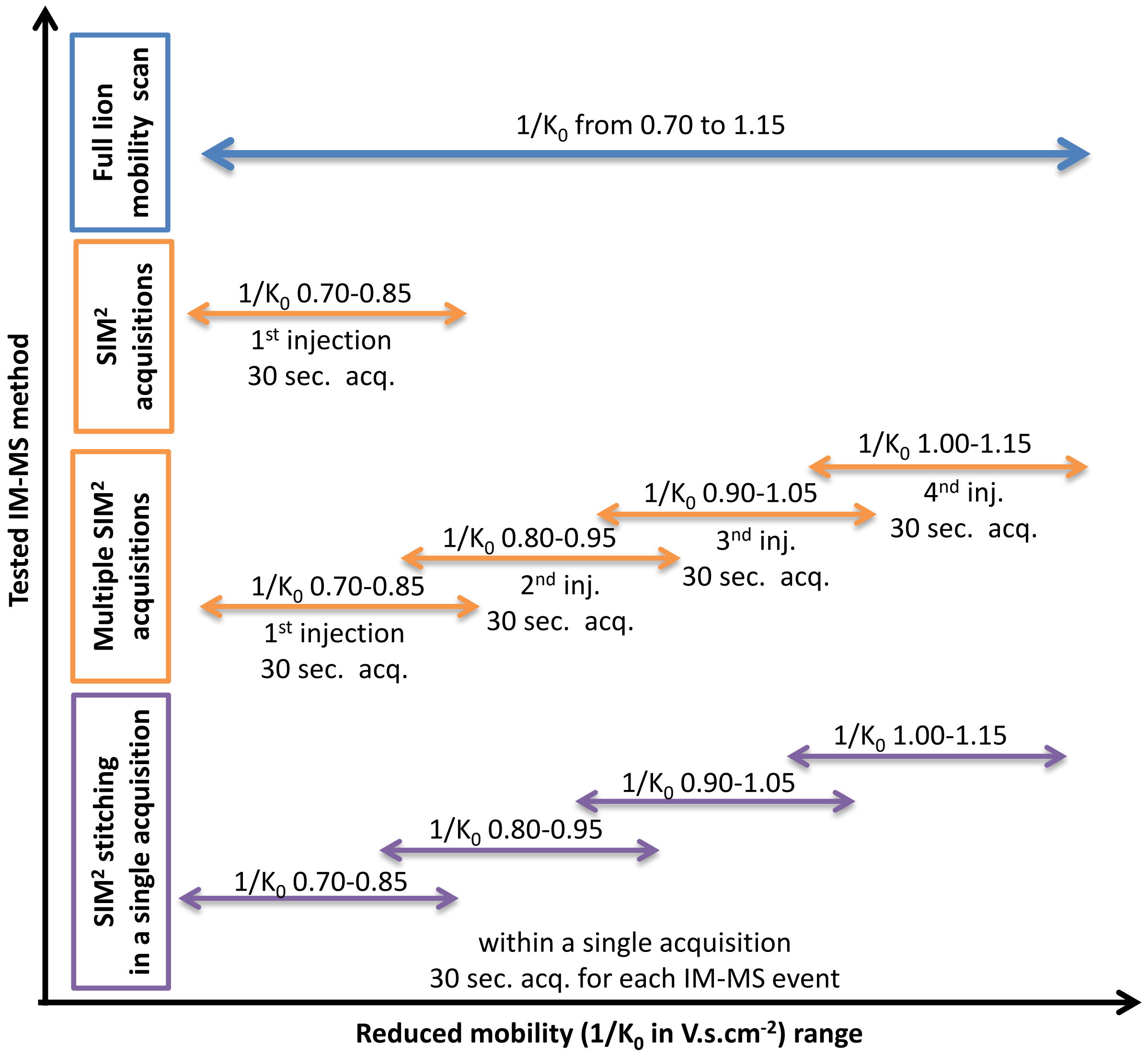

Scheme 1. Schematic description of differents tested IM-MS acqusitions.

Full Ion mobility detection range under moderate IM resolution conditions allows a wide mobility range detection in a single acquisition, in contrast to **SIM^2 method,** for which maximum resolution conditions are reached but in a targeted fashion (see experimental section). Therefore, to obtain a broad IM detection while keeping high resolution conditions, it is possible to perform **serial (multiple) SIM^2 acquisitions** at different mobility ranges under high resolution conditions. An alternative and novel **SIM^2 stitching** is proposed in our study, which consists in the continuous collection of multiple adjacent SIM^2 windows with variable mobility and overlapping ranges. SIM^2 stitching thus allows the coverage a wide mobility range while maintaining at the same time high resolution conditions in a single acquisition.

Fig. S1. Preliminary results from chiral analysis of the ion mobility separations studied in negative and positive ion mode for Phe, Pro and PhG enantiomers. Note that Phenylglycine is a non proteoigenic amino acid used in a previous study, tested as a chiral reference alongside Phe and Pro.

Comparison of positive versus negative mode for chiral IM-MS analysis

Negative mode
[Phe+PhG+Pro-3H+CuII]-

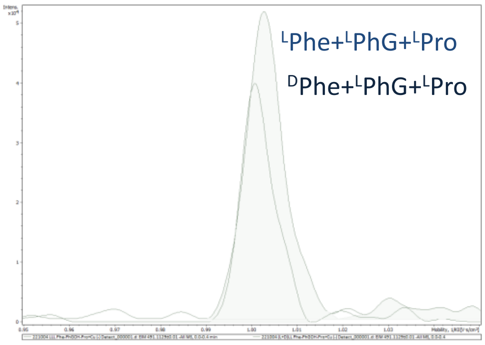

Positive mode
[Phe+PhG+Pro-H+CuII]+

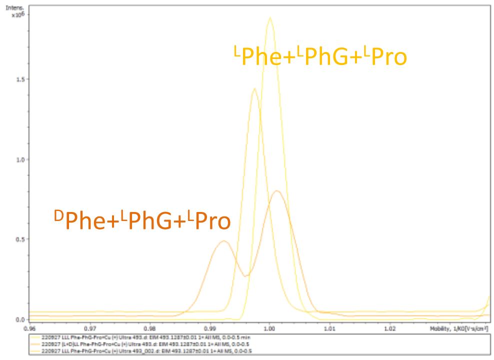

Negative mode
[Phe+Phe+Pro-3H+CuII]-

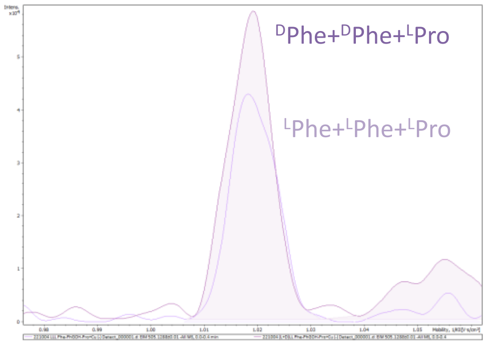

Positive mode
[Phe+Phe+Pro-H+CuII]+

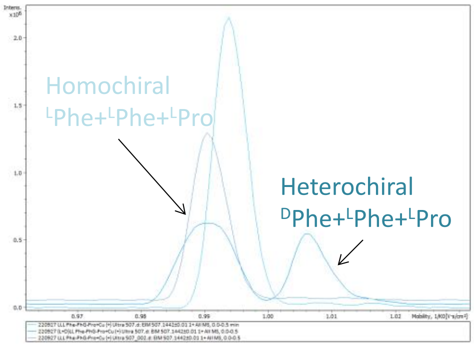

Negative mode
{Phe+PhG+PhG-3H+CuII]-

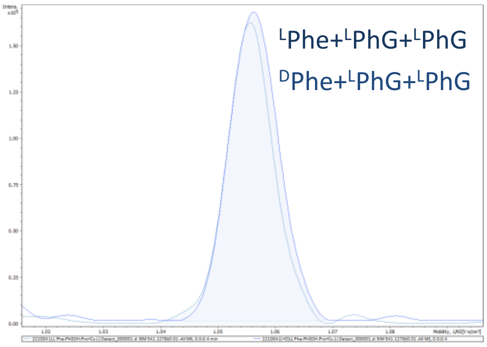

Positive mode
[Phe+PhG+PhG-H+CuII]+

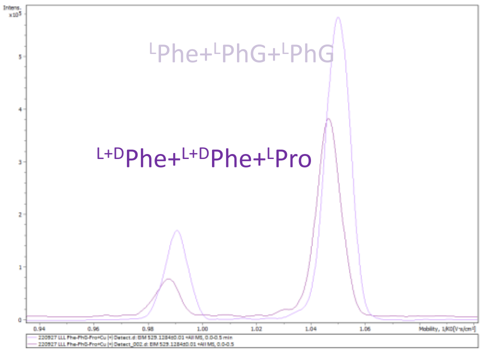

Negative mode
{PhG+PhG+PhG-3H+CuII]-

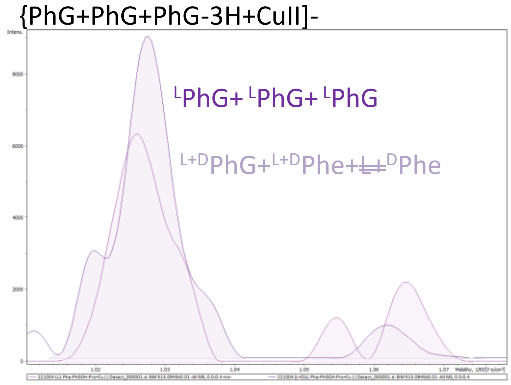

Positive mode
{PhG+PhG+PhG-H+CuII]+

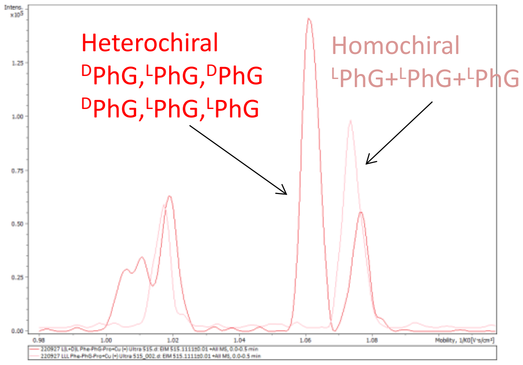

Comparison of chiral analysis from different adduct species detected in positive mode

Positive mode
[PhG+Pro+Pro+Na]+

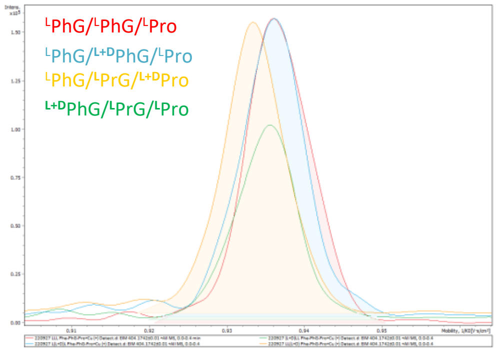

[PhG+Pro+Pro+K]+

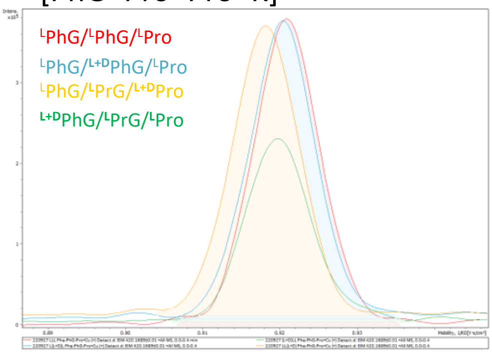

[PhG+Pro+Pro-H+CuII]+

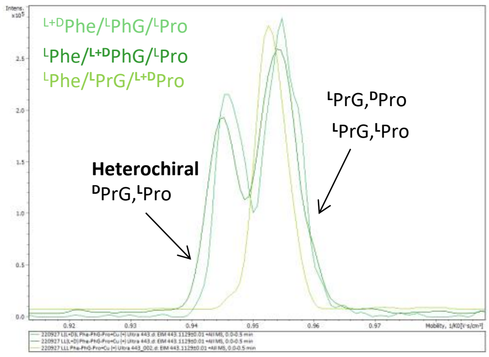

Positive mode
[PhG+PrG+Pro+Na]+

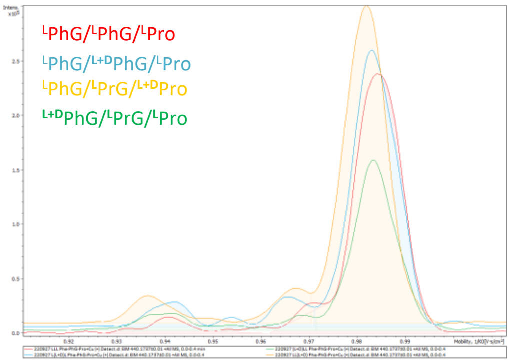

[PhG+PrG+Pro+K]+

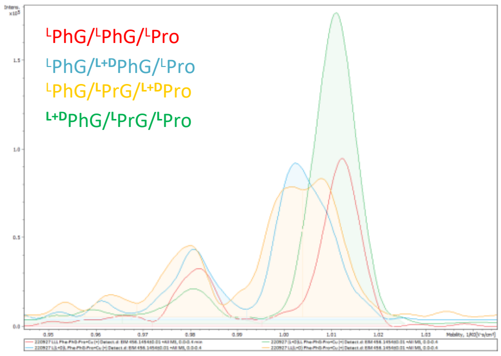

[PhG+PhG+Pro-H+CuII]+

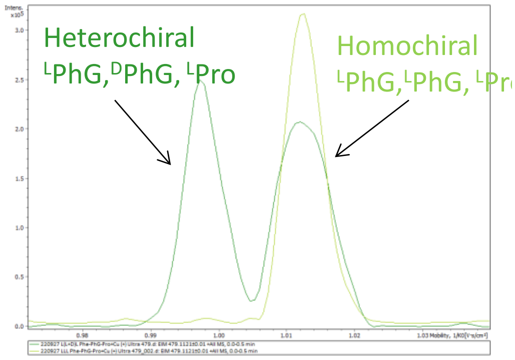

Fig. S2. Examples of the ion mobility separations for all chiral amino acids studied either as dimer and trimer ions. Individual enantiomer solutions (blue and red lines for L and D enantiomer, respectively) are at a concentration of 5 µM whereas equimolar enantiomer mixes (purple line) were at 2.5 µM each (with L-Phe, L-Pro chiral selectors and Cu^{II} always fixed at 5 µM). ΔCCS values (in %) were obtained by calculating the difference between CCS of two isomer peaks in mixture. IM-MS analyses were performed using SIM^2 analysis (see experimental section).

AA	Dimer	Trimer
**Ala**	No ion mobility separation for dimer ions (data not shown)	No ion mobility separation for trimer ions (data not shown)
**Arg**	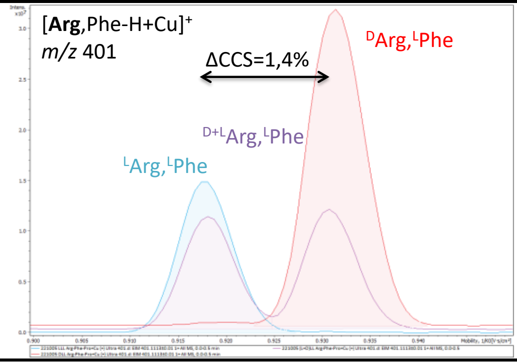	Poor ion mobility separation for trimeric species (data not shown)
**Asn**	Poor ion mobility separation for dimers (data not shown)	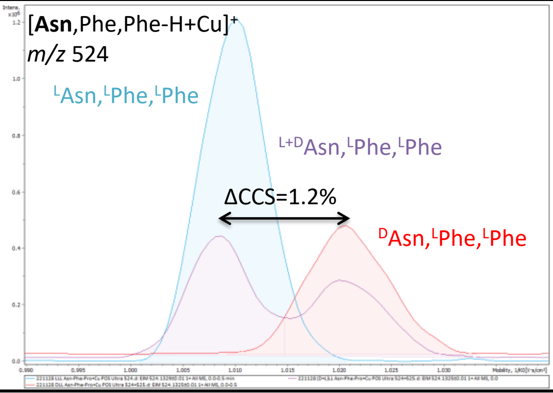
**Asp**	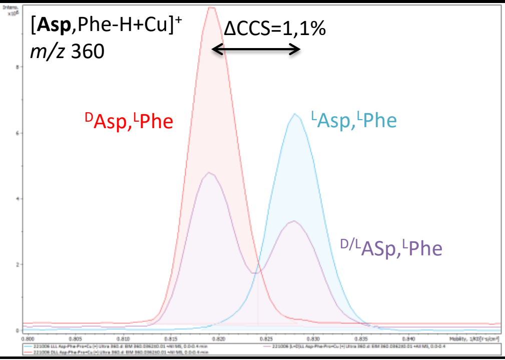	Poor ion mobility separation for trimers (data not shown)

<table>
<tr>
<td>Cys*</td>
<td>Poor ion mobility separation for dimers
(data not shown)</td>
<td>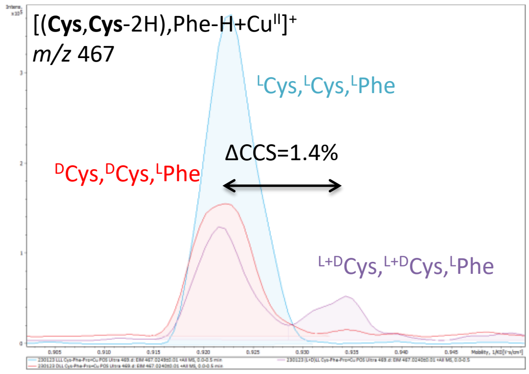

*note that cystine (Cys+Cys-2H) is in fact a covalent dimer</td>
</tr>
<tr>
<td>Gln</td>
<td>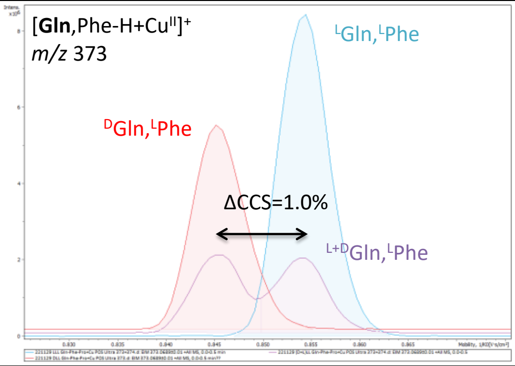
</td>
<td>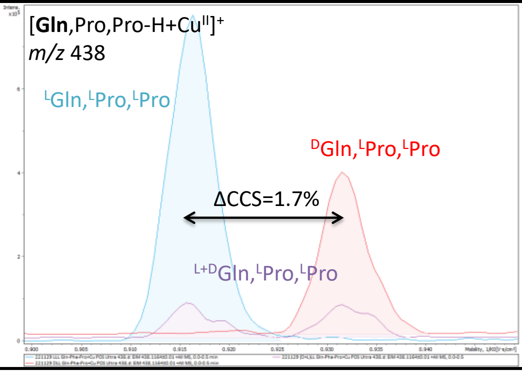
</td>
</tr>
<tr>
<td>Glu</td>
<td>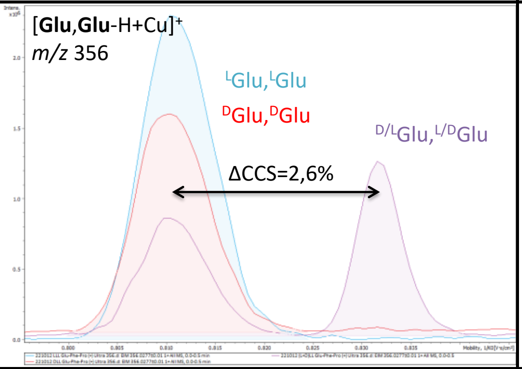
</td>
<td>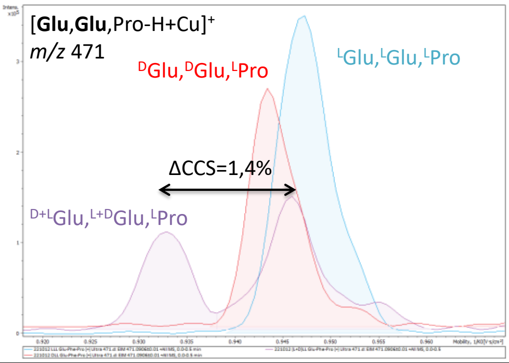
</td>
</tr>
<tr>
<td>His</td>
<td>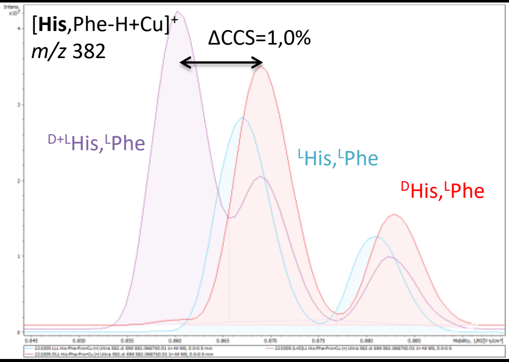
</td>
<td>No trimer ions detected
(data not shown)</td>
</tr>
<tr>
<td>Ile</td>
<td>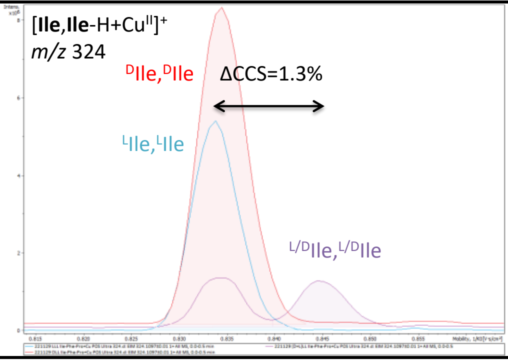
</td>
<td>Poor ion mobility separation for trimers
(data not shown)</td>
</tr>
</table>

<table>
<tr><td>Leu</td><td>Poor ion mobility separation for dimers
(data not shown)</td><td>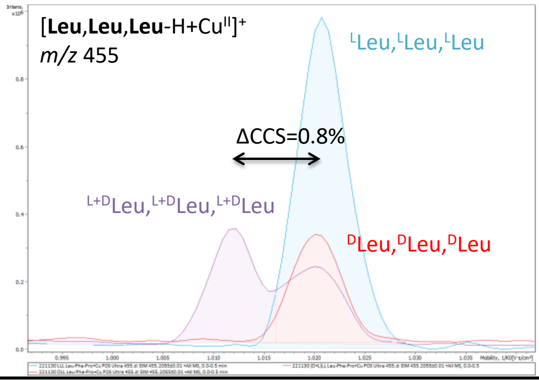
</td></tr>
<tr><td>Lys</td><td>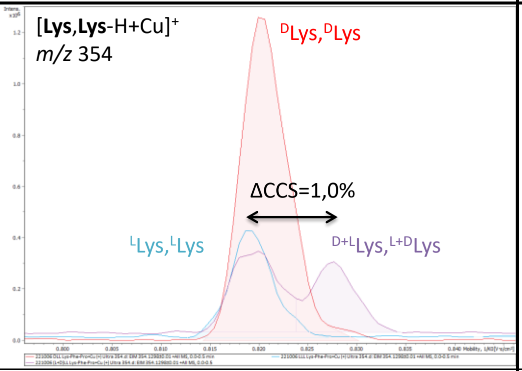
</td><td>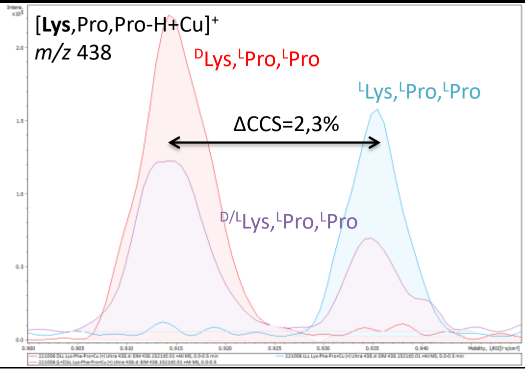
</td></tr>
<tr><td>Met</td><td>Poor ion mobility separation for dimers
(data not shown)</td><td>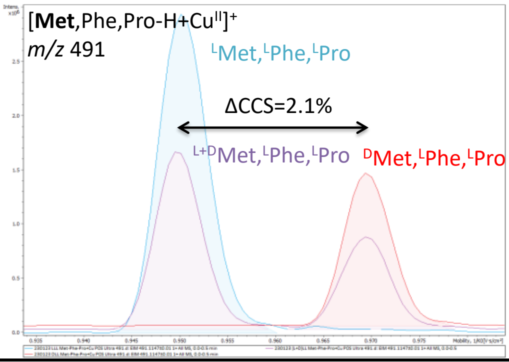
</td></tr>
<tr><td>Phe</td><td>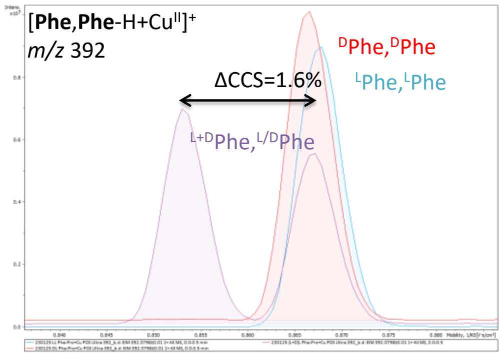
</td><td>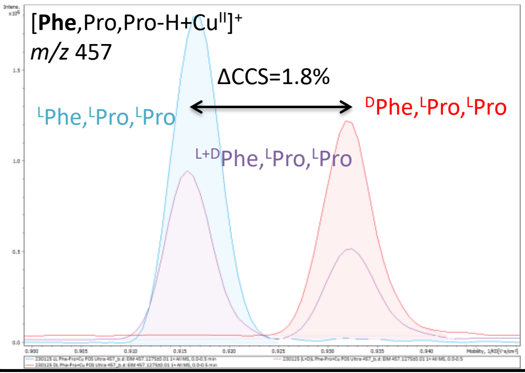
</td></tr>
<tr><td>Pro</td><td>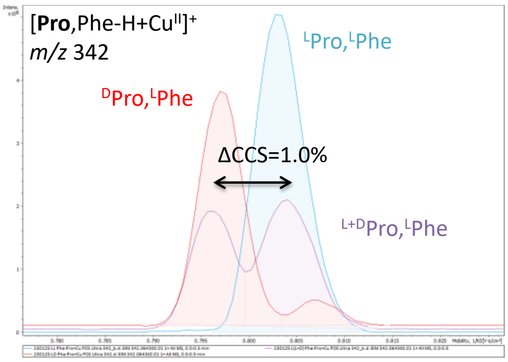
</td><td>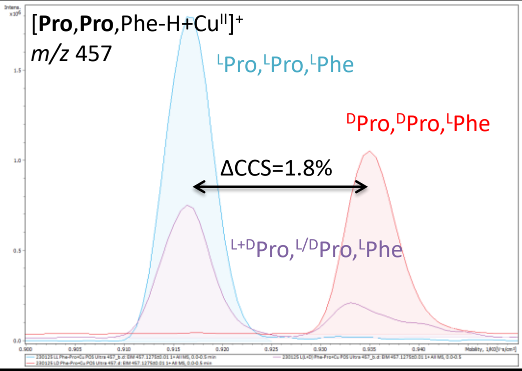
</td></tr>
</table>

Ser

Poor ion mobility separation for dimers (data not shown)

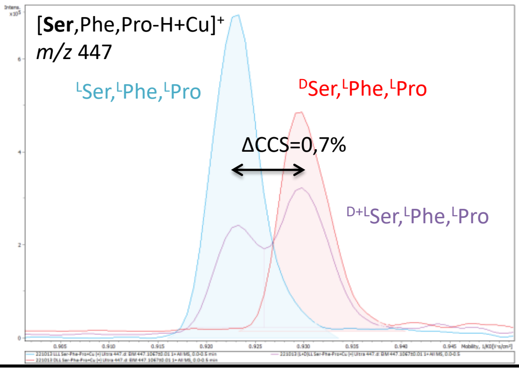

Thr

Poor ion mobility separation for dimers (data not shown)

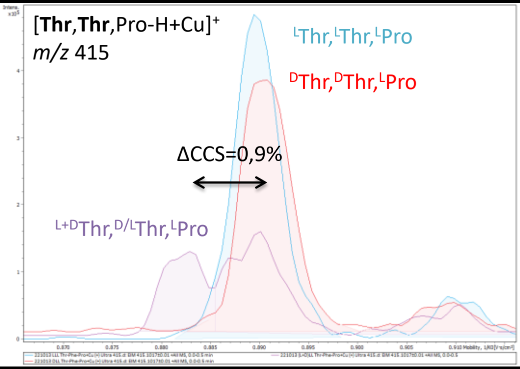

Trp

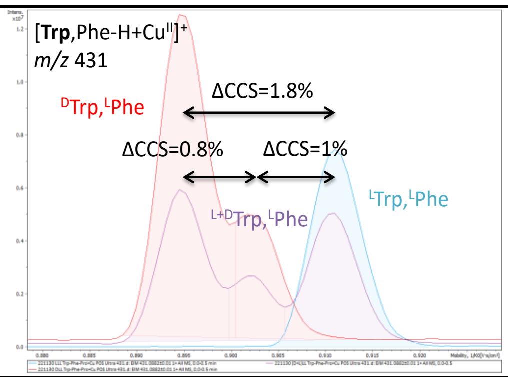

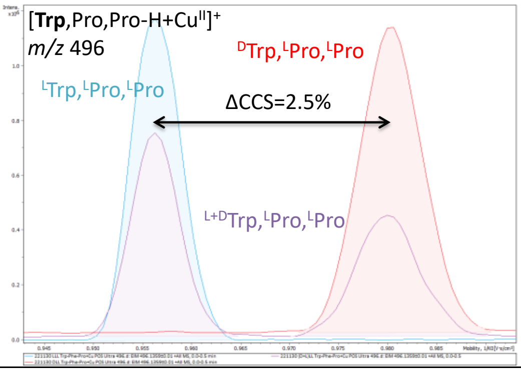

Tyr

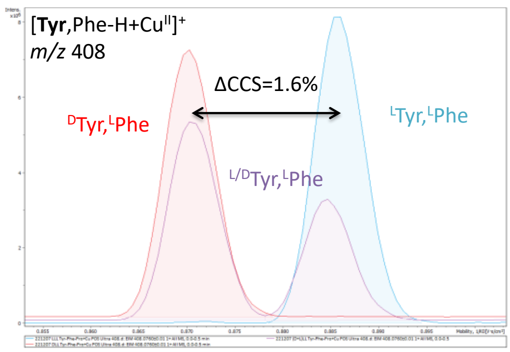

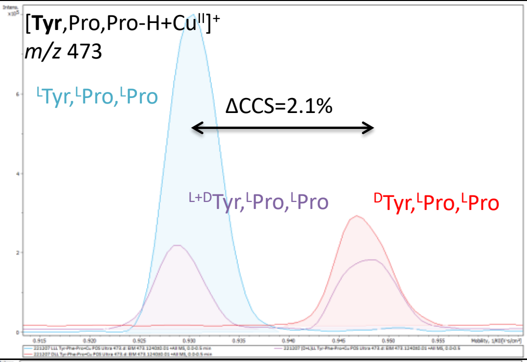

Val

Poor ion mobility separation for dimers (data not shown)

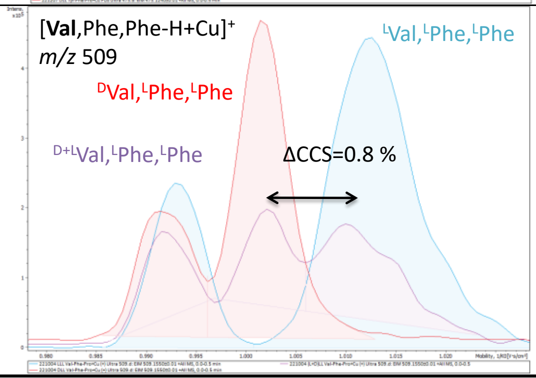

Table S1. CCS (in Å^2) and ΔCCS% values of amino acid enantiomers (2.5 µM each) with L-Phe, L-Pro and Cu^{II} (at 5 µM concentration). Good separations are highlighted in green, partial separations are highlighted in orange and insufficient or absent separations are highlighted in grey. Purple font indicates self-association, i.e. the ion mobility signals do not correspond to L- or D-enantiomer but to heterochiral versus homochiral chiral cluster ions.

Excluding Phe and Pro amino acids (as their L-enantiomers are used as chiral references), among 153 possible complexes, 125 are detected (82%), and 30 of them are separated by ion mobility experiments under SIM^2 mode (24% of detected complexes, 20% of all possible complexes), and 10 are partially resolved.

Complex	AA	*m/z*	CCS ^{L}AA	CCS ^{D}AA	ΔCCS%
AA/Pro	Ala	266.032	153.7		
AA/Phe	Ala	316.048	160.8		
AA/AA	Ala	240.017	147.7		
AA/Pro/Pro	Ala	381.096	176.2		
AA/Phe/Pro	Ala	431.111	183.7		
AA/Phe/Phe	Ala	481.127	195.4		
AA/AA/Pro	Ala	355.080			
AA/AA/Phe	Ala	405.096	188.8		
AA/AA/AA	Ala	329.064			
AA/Pro	Arg	351.096	179.7	182.1	1.3%
AA/Phe	Arg	401.112	190.2	192.8	1.4%
AA/AA	Arg	410.145	187.1	189.2	1.1%
AA/Pro/Pro	Arg	466.160	198.3		
AA/Phe/Pro	Arg	516.173	207.2	209.1	0.9%
AA/Phe/Phe	Arg	566.191	222.6		
AA/AA/Pro	Arg	525.208			
AA/AA/Phe	Arg	575.224			
AA/AA/AA	Arg	584.257			
AA/Pro	Asn	309.038	159.9		
AA/Phe	Asn	359.054	170.9		
AA/AA	Asn	326.028	164.6		
AA/Pro/Pro	Asn	424.101			
AA/Phe/Pro	Asn	474.117	193.0	193.9	0.5%
AA/Phe/Phe	Asn	524.133	207.3	209.7	1.2%
AA/AA/Pro	Asn	441.092	186.6		
AA/AA/Phe	Asn	491.107			
AA/AA/AA	Asn	458.082			
AA/Pro	Asp	310.023	159.6		
AA/Phe	Asp	360.037	172.1	170.3	1.1%
AA/AA	Asp	327.996	163.7		
AA/Pro/Pro	Asp	425.086	183.9		
AA/Phe/Pro	Asp	475.101	193.3		
AA/Phe/Phe	Asp	525.117	206.2		
AA/AA/Pro	Asp	443.060			
AA/AA/Phe	Asp	493.075			
AA/AA/AA	Asp	461.034			

AA/Pro	Cys	298.004			
AA/Phe	Cys	348.020			
AA/AA	Cys	301.945	155.4		
AA/Pro/Pro	Cys	413.068			
AA/Phe/Pro	Cys	463.083			
AA/Phe/Phe	Cys	513.099			
AA/AA/Pro	Cys	417.008	175.9		
AA/AA/Phe	Cys	467.024	190.0	192.6	1.4%
AA/AA/AA	Cys	424.981			
AA/Pro	Gln	323.054	164.5		
AA/Phe	Gln	373.069	177.3	175.5	1.0%
AA/AA	Gln	354.060	173.7		
AA/Pro/Pro	Gln	438.117	189.2	192.4	1.7%
491	Gln	488.133	197.6	199.9	1.2%
AA/Phe/Phe	Gln	538.148	210.1		
AA/AA/Pro	Gln	469.123	194.8		
AA/AA/Phe	Gln	519.138	206.7	208.9	1.1%
AA/AA/AA	Gln	500.129	203.6		
AA/Pro	Glu	324.037	166.7		
AA/Phe	Glu	374.054	177.7	176.2	0.8%
AA/AA	Glu	356.027	168.6	173.0	2.6%
AA/Pro/Pro	Glu	439.101	188.8		
AA/Phe/Pro	Glu	489.117	199.2		
AA/Phe/Phe	Glu	539.132	211.1		
AA/AA/Pro	Glu	471.091	192.3	195.0	1.4%
AA/AA/Phe	Glu	521.107	206.9		
AA/AA/AA	Glu	503.081	201.1		
AA/Pro	His	332.054	171.1		
AA/Phe	His	382.070	178.5	180.3	1.0%
AA/AA	His	372.060	177.9		
AA/Pro/Pro	His	447.118			
AA/Phe/Pro	His	497.133			
AA/Phe/Phe	His	547.149			
AA/AA/Pro	His	487.124			
AA/AA/Phe	His	537.139			
AA/AA/AA	His	527.130			
AA/Pro	Ile	308.079	167.8	168.5	0.4%
AA/Phe	Ile	358.095	175.0		
AA/AA	Ile	324.111	174.1	176.3	1.3%
AA/Pro/Pro	Ile	423.143	191.5		
AA/Phe/Pro	Ile	473.158	198.7		
AA/Phe/Phe	Ile	523.174	210.6	208.7	0.9%
AA/AA/Pro	Ile	439.174	198.8		
AA/AA/Phe	Ile	489.190	206.9		
AA/AA/AA	Ile	455.205	205.8		

AA/Pro	Leu	308.079	171.2		
AA/Phe	Leu	358.095	176.4		
AA/AA	Leu	324.111	179.8	180.9	0.6%
AA/Pro/Pro	Leu	423.143	194.0		
AA/Phe/Pro	Leu	473.158	200.9		
AA/Phe/Phe	Leu	523.174	210.7		
AA/AA/Pro	Leu	439.174	202.9		
AA/AA/Phe	Leu	489.190	209.5		
AA/AA/AA	Leu	455.205	208.8	210.4	0.8%
AA/Pro	Lys	323.091	175.1		
AA/Phe	Lys	373.106	183.8		
AA/AA	Lys	354.132	170.4	172.1	1.0%
AA/Pro/Pro	Lys	438.153	193.1	188.8	2.3%
AA/Phe/Pro	Lys	488.170	200.4		
AA/Phe/Phe	Lys	538.185	208.8	210.0	0.6%
AA/AA/Pro	Lys	469.196			
AA/AA/Phe	Lys	519.212			
AA/AA/AA	Lys	500.238			
AA/Pro	Met	326.036	164.1		
AA/Phe	Met	376.051	174.3	173.1	0.7%
AA/AA	Met	360.023	169.7		
AA/Pro/Pro	Met	441.099	187.0		
AA/Phe/Pro	Met	491.115	195.5	199.6	2.1%
AA/Phe/Phe	Met	541.131	211.1		
AA/AA/Pro	Met	475.087	194.4		
AA/AA/Phe	Met	525.102	206.5	209.4	1.4%
AA/AA/AA	Met	509.074	204.0		
AA/Pro	Phe	342.064	167.1	165.5	1.0%
AA/Phe	Phe				
AA/AA	Phe	392.079	179.7	176.9	1.6%
AA/Pro/Pro	Phe	457.127	188.9	192.3	1.8%
AA/Phe/Pro	Phe				
AA/Phe/Phe	Phe				
AA/AA/Pro	Phe	507.143	200.1	203.0	1.4%
AA/AA/Phe	Phe				
AA/AA/AA	Phe	557.159	215.4		
AA/Pro	Pro				
AA/Phe	Pro	342.064	167.4	165.8	1.0%
AA/AA	Pro	292.048	160.1		
AA/Pro/Pro	Pro				
AA/Phe/Pro	Pro				
AA/Phe/Phe	Pro	507.143	200.3		
AA/AA/Pro	Pro				
AA/AA/Phe	Pro	457.127	189.1	192.5	1.8%
AA/AA/AA	Pro	407.111	183.4		

AA/Pro	Ser	282.027	158.2		
AA/Phe	Ser	332.043	167.8		
AA/AA	Ser	272.006	157.7		
AA/Pro/Pro	Ser	397.090	185.1		
AA/Phe/Pro	Ser	447.106	190.6	191.9	0.7%
AA/Phe/Phe	Ser	497.122	205.7	207.2	0.7%
AA/AA/Pro	Ser	387.070	176.5		
AA/AA/Phe	Ser	437.085	191.2	192.2	0.5%
AA/AA/AA	Ser	377.049			
AA/Pro	Thr	296.043	161.8		
AA/Phe	Thr	346.059	170.8		
AA/AA	Thr	300.038	163.6		
AA/Pro/Pro	Thr	411.106	182.5	181.7	0.4%
AA/Phe/Pro	Thr	461.122	194.1		
AA/Phe/Phe	Thr	511.137	208.9		
AA/AA/Pro	Thr	415.101	182.5	184.1	0.9%
AA/AA/Phe	Thr	465.117	197.4		
AA/AA/AA	Thr	419.096	185.3		
AA/Pro	Trp	381.074	174.3		
AA/Phe	Trp	431.090	188.2	184.9	1.8%
AA/AA	Trp	470.101	192.1		
AA/Pro/Pro	Trp	496.138	196.8	201.7	2.5%
AA/Phe/Pro	Trp	546.153	207.8	212.1	2.0%
AA/Phe/Phe	Trp	596.169	223.0	225.9	1.3%
AA/AA/Pro	Trp	585.164	213.9	217.7	1.8%
AA/AA/Phe	Trp	635.180	228.9		
AA/AA/AA	Trp	674.191	232.4		
AA/Pro	Tyr	358.059	169.3		
AA/Phe	Tyr	408.074	183.1	180.2	1.6%
AA/AA	Tyr	424.069	183.6	186.4	1.5%
AA/Pro/Pro	Tyr	473.122	191.4	195.4	2.1%
AA/Phe/Pro	Tyr	523.137	203.5	206.2	1.3%
AA/Phe/Phe	Tyr	573.153	219.6		
AA/AA/Pro	Tyr	539.132	206.1	209.3	1.5%
AA/AA/Phe	Tyr	589.148	221.3	222.5	0.5%
AA/AA/AA	Tyr	605.143	224.8		
AA/Pro	Val	294.064	165.5		
AA/Phe	Val	344.079	172.4		
AA/AA	Val	296.079	167.1	168.2	0.7%
AA/Pro/Pro	Val	409.127	188.6		
AA/Phe/Pro	Val	459.143	195.8		
AA/Phe/Phe	Val	509.159	207.8	206.1	0.8%
AA/AA/Pro	Val	411.143	191.8		
AA/AA/Phe	Val	461.158	195.7		
AA/AA/AA	Val	413.158			

Fig. S3. Extracted ion mobility signals at *m/z* 324 ([AA,AA-H+Cu^{II}]$^+$) of various mixes of L and/or D-Ile and Leu isomers. IM-MS analyses were performed using SIM^2 analysis (see experimental section).

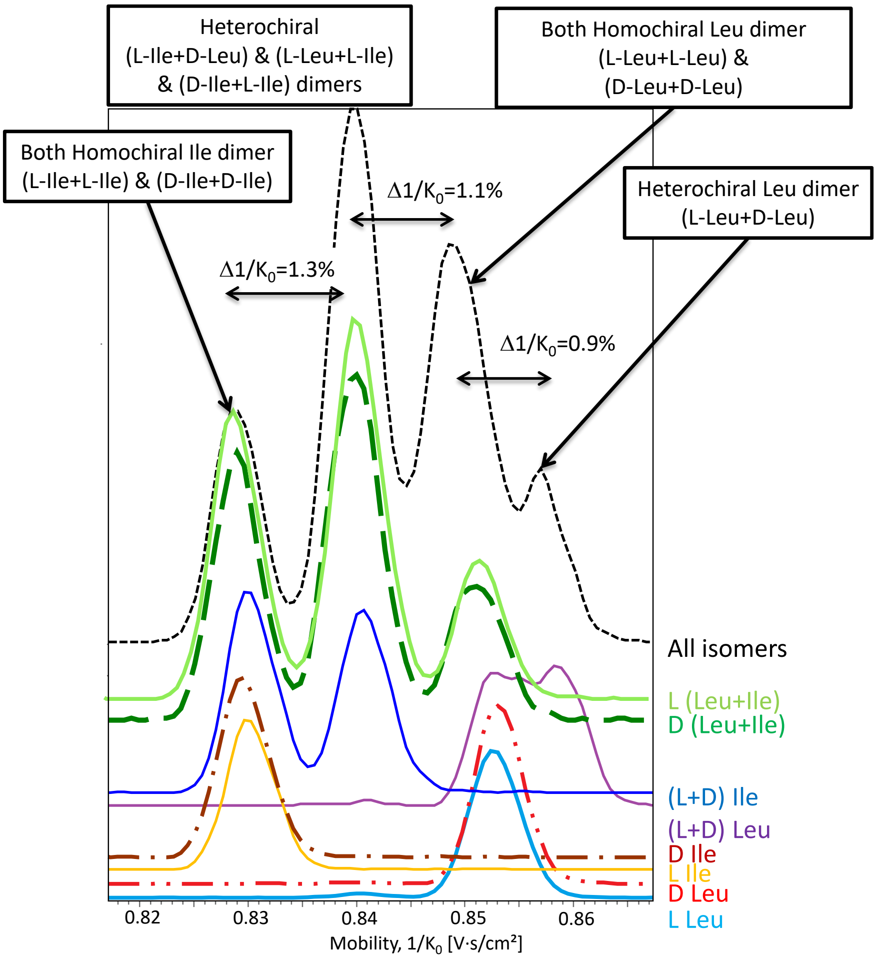

Table S2. Ion mobility separations obtained for L and D Leu and Ile isomers using SIM^2 analysis of equimolar isomer mixes, expressed as ΔCCS% values.

	L versus D		Leu vs Ile		All isomers (L&D Leu+Ile)		
	Leu only	Ile only	L only	D only			
**AA**,Pro	-	-	1.4%	1.1%	0.6%	0.8%	
**AA**,Phe	-	-	-	0.8%		-	
**AA**,**AA**	0.6%	1.3%	1.4%/1.3%	1.3%/1.3%	1.3%	1.1%	0.9%
**AA**,Pro,Pro	-	-	0.9%	-		0.8%	
**AA**,Phe,Pro	-	-	0.6%	-		-	
**AA**,Phe,Phe	-	0.9%	-	-		-	
**AA**,**AA**,Pro	-	-	0.8%/-	0.8%/-		-	
**AA**,**AA**,Phe	-	-	-	-		-	
**AA**,**AA**,**AA**	0.8%	-	-	-		-	

(-) Ions were detected but ΔCCS% value too low for proper ion mobility separation (ΔCCS% < 0.5%)

Fig. S4. Multiple SIM^2 analysis (mobility window width of 0.10 V.s/cm^2) of a mix of L- and D-Arg, Gln, Met, Trp and Tyr (each enantiomer at 1 µM) with increasing amounts of Cu^{II}, L-Phe and L-Pro (left, 1 to 10 µM) and increasing amounts of L-Phe and L-Pro with 10 µM of Cu^{II} (right, 1 to 10 µM).

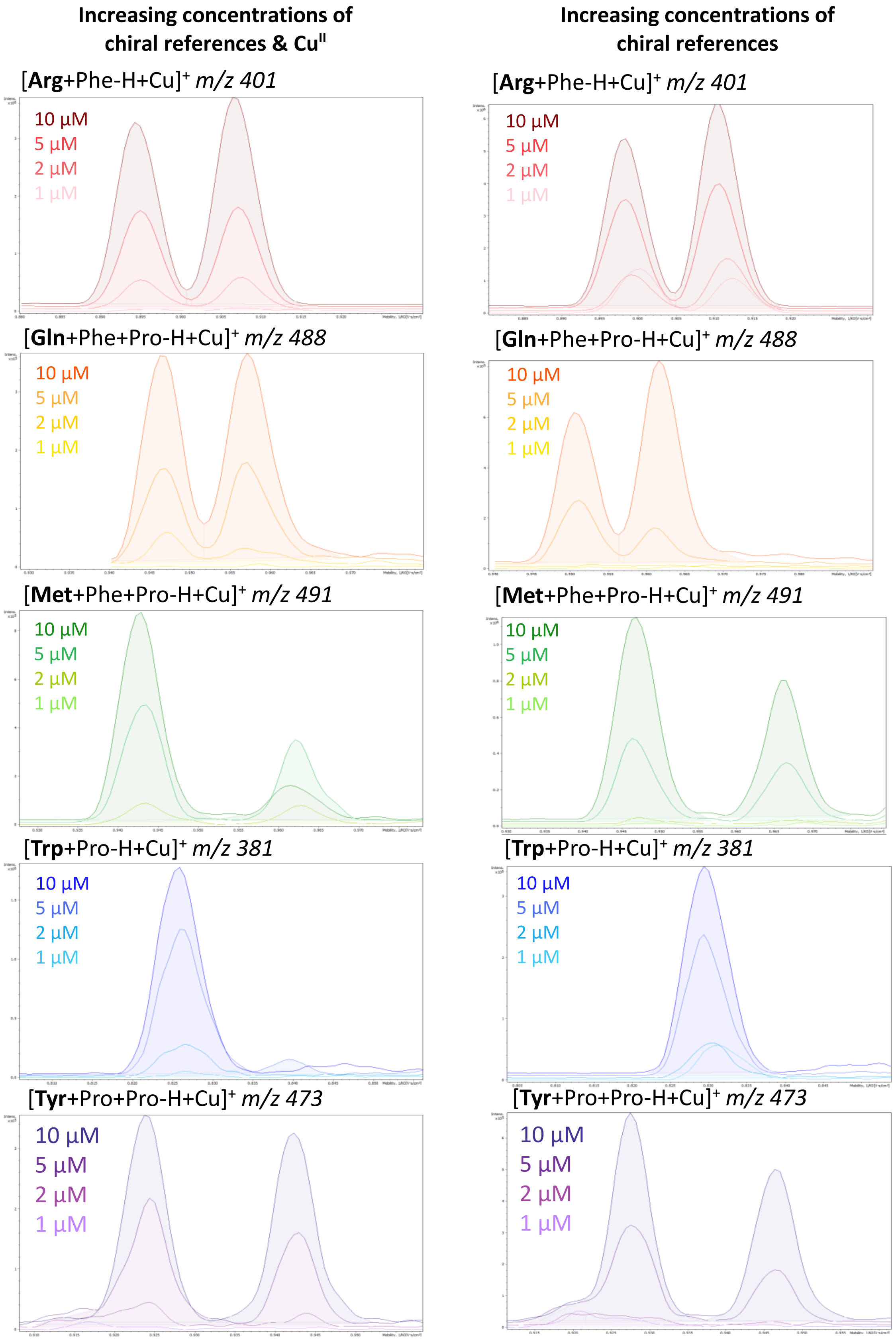

Fig. S5. Multiple SIM^2 analysis for the mobility separation of an equimolar mixture of Arg, Asp, Cys, Gln, Ile, Ser, Trp and Val enantiomers with fixed reduced mobility ($1/K_0$) window widths of 0.10 V.s.cm^{-2} (i.e. ranges of 0.70-0.80, 0.76-0.86, 0.82-0.92, 0.88-0.98, 0.94-1.04, 1.00-1.10, 1.06-1.16 ranges, lighter), widths of 0.15 V.s.cm^{-2} (0.70-0.85, 0.80-0.95, 0.90-1.05, 1.00-1.15 ranges) and widths of 0.20 V.s.cm^{-2} (0.70-0.90, 0.85-1.05, 1.00-1.20 ranges, darker) see **Scheme 1**. Each AA enantiomer was at a concentration of 1 µM, and L-Phe, L-Pro chiral references and Cu^{II} at 20 µM.

(a) Stacked view of ion mobility spectra for various coppered complex ions

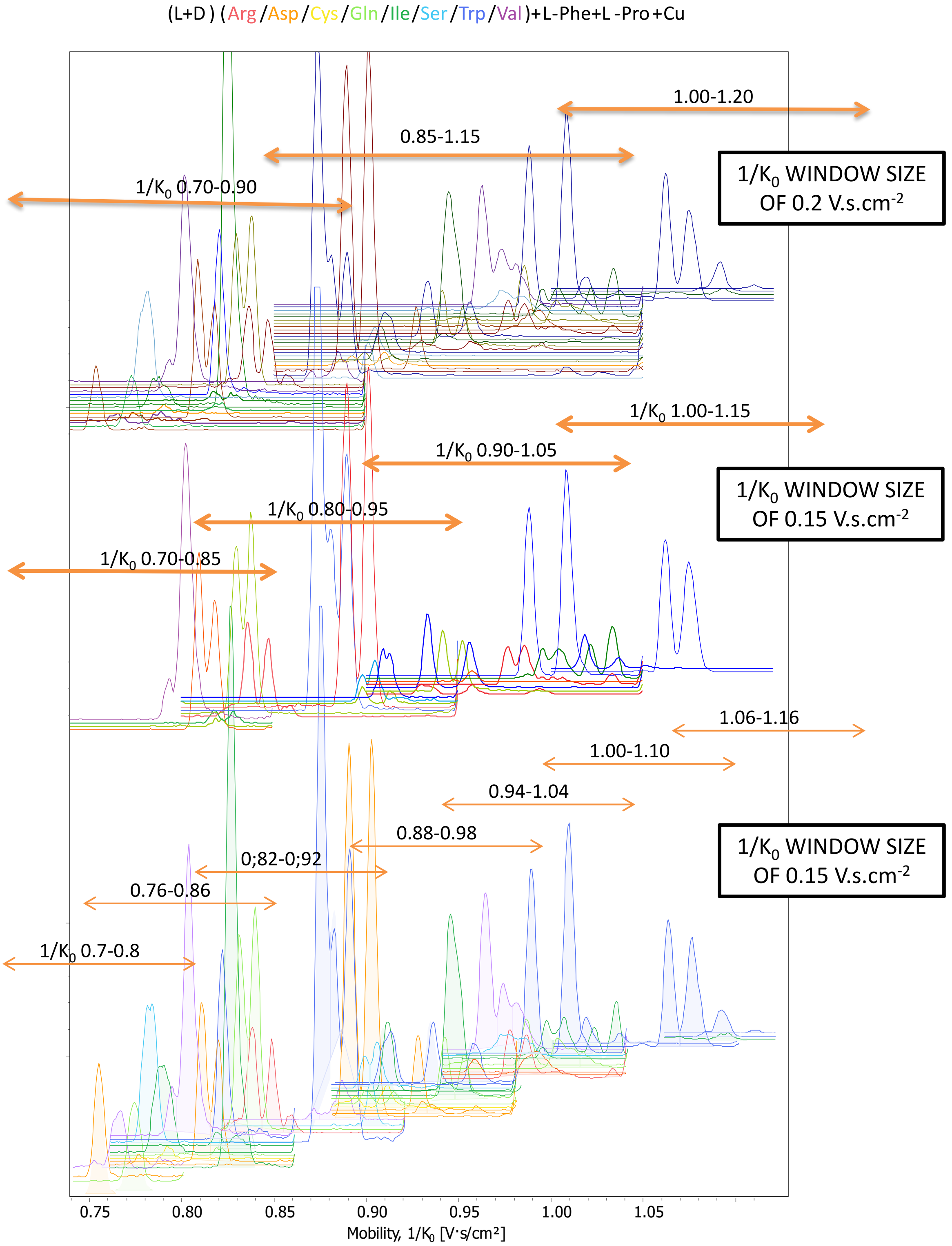

Fig. S5 (continued). Multiple SIM^2 analysis for the mobility separation of an equimolar mixture of Arg, Asp, Cys, Gln, Ile, Ser, Trp and Val enantiomers with fixed reduced mobility ($1/K_0$) window widths of 0.10 V.s.cm^{-2} (i.e. ranges of 0.70-0.80, 0.76-0.86, 0.82-0.92, 0.88-0.98, 0.94-1.04, 1.00-1.10, 1.06-1.16 ranges, lighter), widths of 0.15 V.s.cm^{-2} (0.70-0.85, 0.80-0.95, 0.90-1.05, 1.00-1.15 ranges) and widths of 0.20 V.s.cm^{-2} (0.70-0.90, 0.85-1.05, 1.00-1.20 ranges, darker) see **Scheme 1**. Each AA enantiomer was at a concentration of 1 µM, and L-Phe, L-Pro chiral references and Cu^{II} at 20 µM.

(b) Overlaid views of the previously reported signals

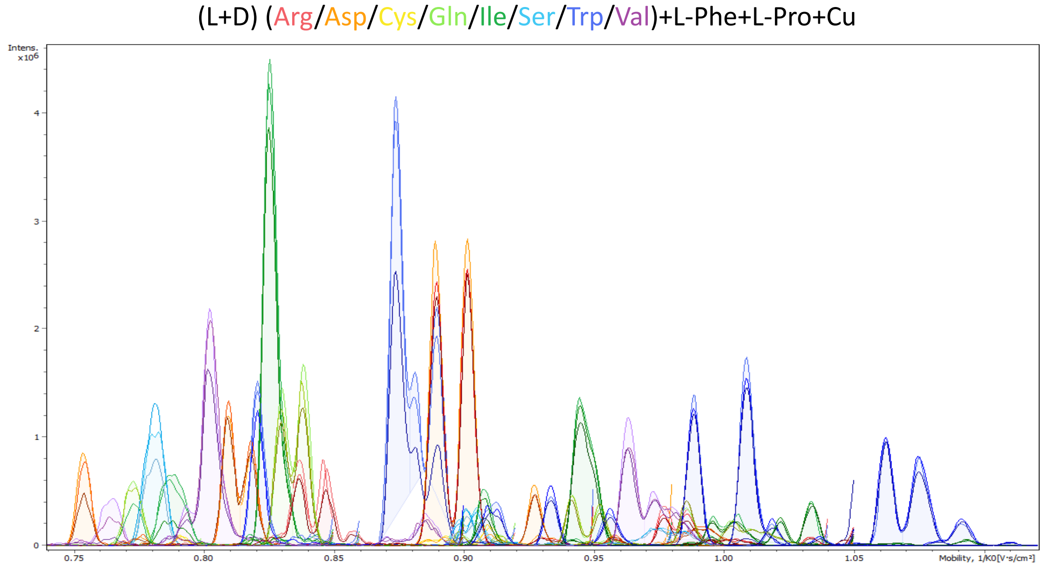

(c) Zooms of overlaid views

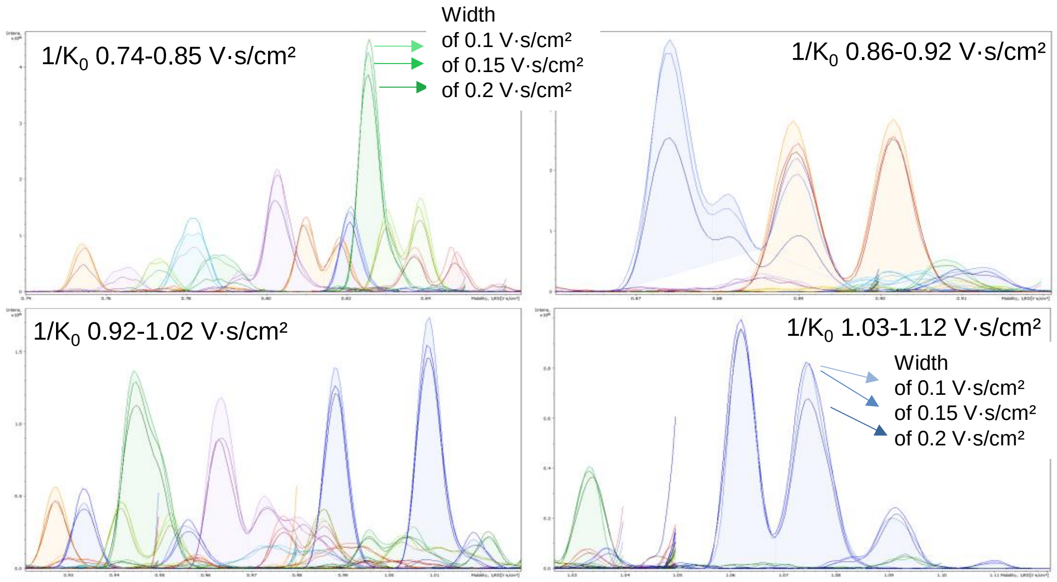

Fig. S6. Multiple SIM² analysis of an equimolar mix of Asn, Glu, His, Leu, Lys, Met, Thr and Tyr enantiomers using fixed mobility window widths of 0.10 V.s.cm^{-2} (i.e. ranges of 0.70-0.80, 0.76-0.86, 0.82-0.92, 0.88-0.98, 0.94-1.04, 1.00-1.10, 1.06-1.16 V.s.cm^{-2}, lighter), 0.15 V.s.cm^{-2} (0.70-0.85, 0.80-0.95, 0.90-1.05, 1.00-1.15 V.s.cm^{-2}) and 0.20 V.s.cm^{-2} (0.70-0.90, 0.85-1.05, 1.00-1.20 V.s/cm², darker) see **Scheme 1**. Each AA enantiomer was at a concentration of 1 µM, L-Phe, L-Pro chiral references and Cu^{II} at 20 µM.

a) Stacked view of ion mobility spectra for various coppered complex ions

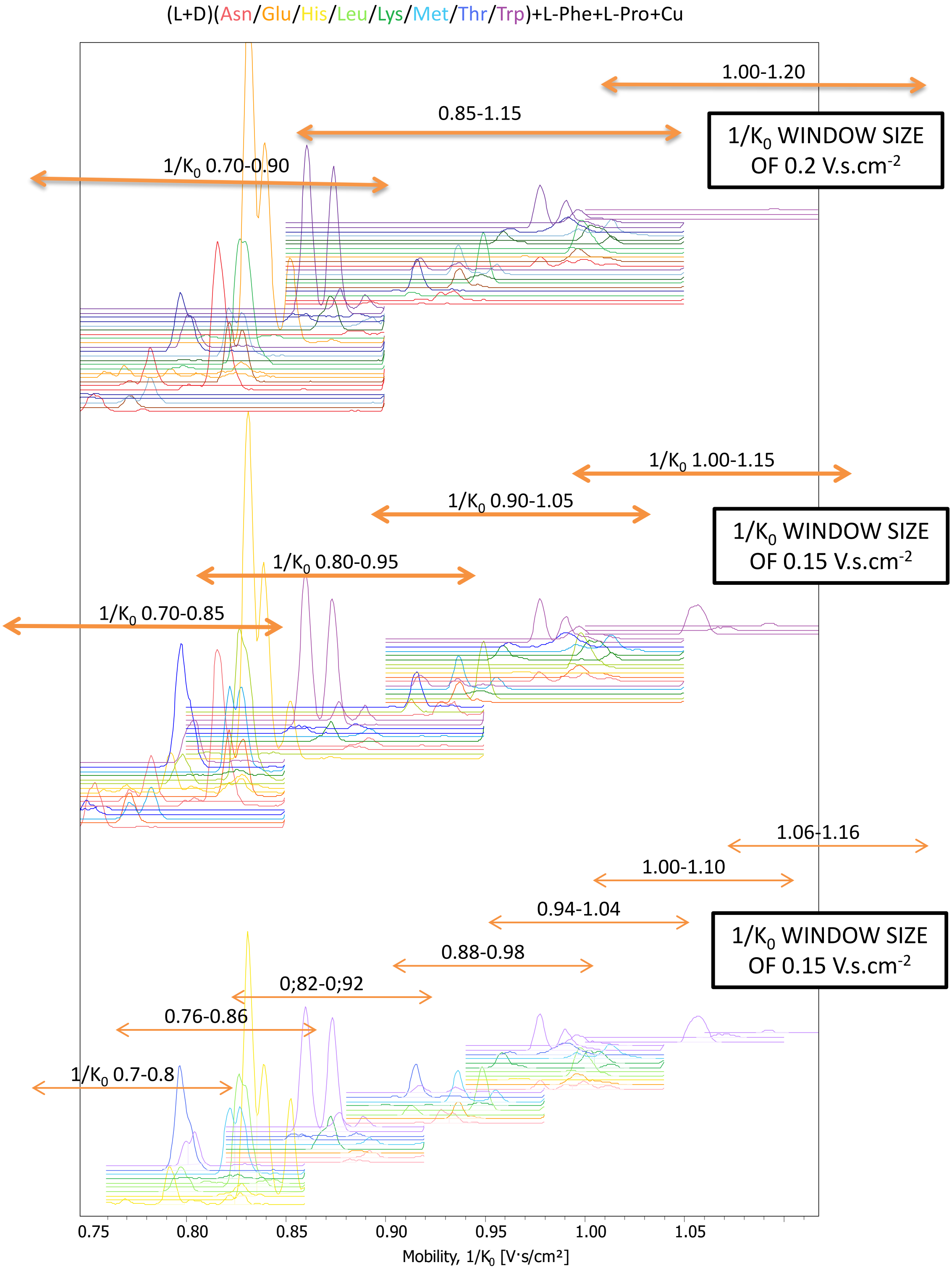

Fig. S6 (continued). Multiple SIM^2 analysis of an equimolar mix of Asn, Glu, His, Leu, Lys, Met, Thr and Tyr enantiomers using fixed mobility window widths of 0.10 V.s.cm^{-2} (i.e. ranges of 0.70-0.80, 0.76-0.86, 0.82-0.92, 0.88-0.98, 0.94-1.04, 1.00-1.10, 1.06-1.16 V.s.cm^{-2}, lighter), 0.15 V.s.cm^{-2} (0.70-0.85, 0.80-0.95, 0.90-1.05, 1.00-1.15 V.s.cm^{-2}) and 0.20 V.s.cm^{-2} (0.70-0.90, 0.85-1.05, 1.00-1.20 V.s/cm^2, darker) see **Scheme 1** below. Each AA enantiomer was at a concentration of 1 µM, L-Phe, L-Pro chiral references and Cu^{II} at 20 µM.

(b) Overlaid views of the previously reported signals

(L+D)(Asn/Glu/His/Leu/Lys/Met/Thr/Trp)+L-Phe+L-Pro+Cu

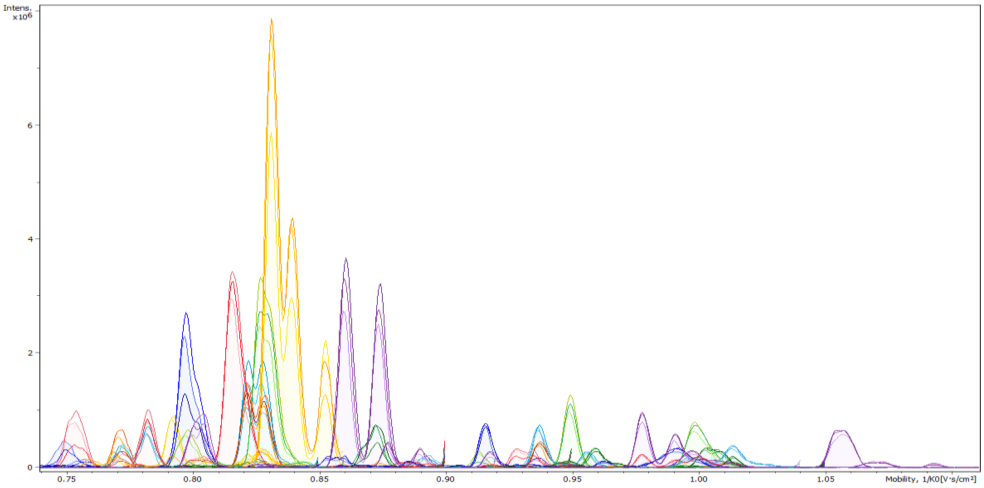

(c) Zooms of overlaid views

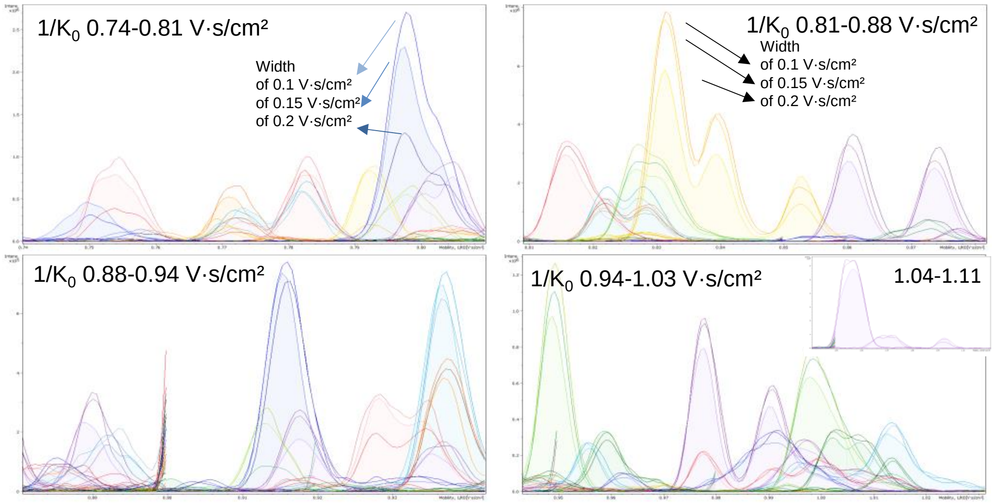

Table S3. Results from the SIM^2 stitching analysis of an enantiomeric mix of Arg, Asp, Cys, Gln, Ile, Ser, Trp and Val (1 µM each), L-Phe and L-Pro (10 µM each) and Cu^{II} (20 µM).

[...-H+Cu^{II}]$^+$	Arg	Asp	Cys*	Gln	Ile	Ser	Trp	Val
**AA**,Pro	✓	-	x	-	-	-	-	-
**AA**,Phe	✓	✓	x	✓	-	-	✓	-
**AA**,**AA**	x	-	-	-	✓	-	-	x
**AA**,Pro,Pro	x	x	x	✓	-	-	✓	-
**AA**,Phe,Pro	-	-	x	✓	-	-	✓	-
**AA**,Phe,Phe	x	-	x	x	✓	x	✓	x
**AA**,**AA**,Pro	x	x	x	x	-	-	✓	x
**AA**,**AA**,Phe	x	x	-	x	-	-	-	x
**AA**,**AA**,**AA**	x	x	x	x	-	x	x	x

(x) not detected;
(-) Ions are detected but either there is no separation of enantiomers or ΔCCS% values are too low for proper separation (typically ΔCCS% < 0.5%);
✓ Separation between L and D enantiomers
* The ions detected for Cys correspond to its reduced dimer, *i.e.*, [(Cys,Cys-2H),ref-H+Cu^{II}]$^+$

Among 72 possible copper complex species with Arg, Asp, Cys, Gln, Ile, Ser, Trp and Val enantiomers, 43 are detected (60%), from which 13 are separated SIM^2 experiments (30%, 18% of total).

Table S4. Results from the SIM^2 stitching analysis of an enantiomeric mix of Ala, Asn, Glu, His, Leu, Lys, Met, Thr and Tyr (1 µM each), L-Phe and L-Pro (10 µM each) and Cu^{II} (20 µM).

[...-H+Cu^{II}]$^+$	Ala	Asn	Glu	His	Leu	Lys	Met	Thr	Tyr
**AA**,Pro	-	-	-	x	-	x	-	-	-
**AA**,Phe	-	-	✓	✓	-	-	x	-	✓
**AA**,**AA**	x	-	✓	-	-	x	-	-	✓
**AA**,Pro,Pro	-	-	-	x	-	x	-	-	✓
**AA**,Phe,Pro	-	-	-	x	-	-	✓	-	✓
**AA**,Phe,Phe	-	x	x	x	-	-	-	-	-
**AA**,**AA**,Pro	x	x	x	x	x	-	x	x	-
**AA**,**AA**,Phe	-	x	x	x	-	x	x	x	-
**AA**,**AA**,**AA**	x	x	x	x	x	x	x	x	x

(x) Ions are not detected;
(-) Ions are detected but either there is no separation of enantiomers or ΔCCS% values are too low for proper separation (typically ΔCCS% < 0.5%);
✓ Separation between L and D enantiomers

Among all 81 possible copper complex species with Ala, Asn, Glu, His, Leu, Lys, Met, Thr and Tyr enantiomers, 48 are detected (58%), from which 8 are separated by SIM^2 experiments (17%, 10% of total).

Fig. S7. Extracted ion mobility spectra of the complex ions detected from the SIM² stitching analysis of an enantiomeric mix of Arg, Asp, Cys, Gln, Ile, Ser, Trp and Val (1 µM each), L-Phe and L-Pro (10 µM each) and Cu^{II} (20 µM). Note that the reported ion mobility signals are extracted only from a mobility segment that include each ion of interest (either 0.70-0.85, 0.80-0.95, 0.90-1.05 or 1.00-1.15 V.s/cm², **Scheme S1**) in contrast to **Fig. S8** where the same ion mobility spectra at given *m/z* ratio are averaged onto the entire acquisition (including the four ion mobility range segments).

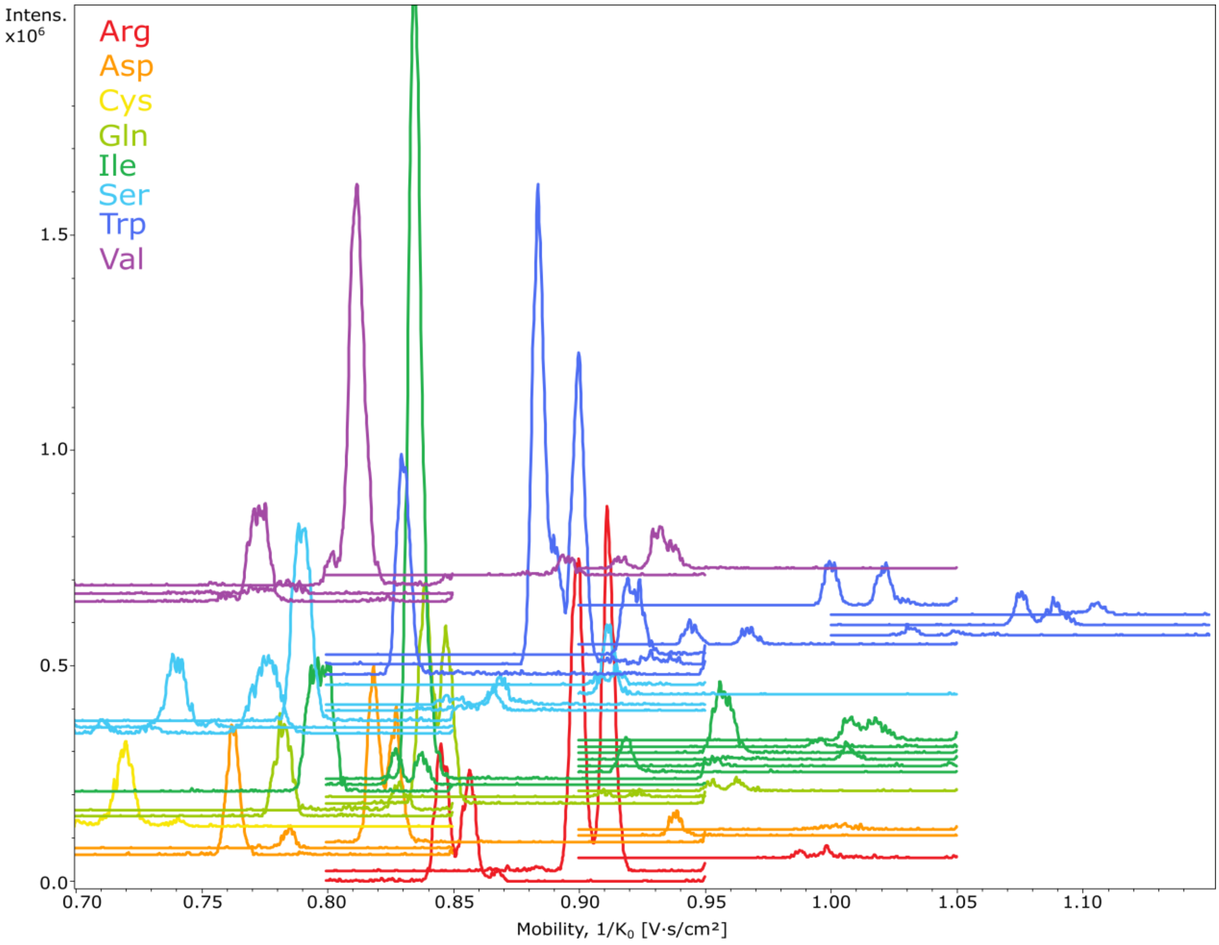

Fig. S8. Extracted ion mobility spectra of the ions detected from the SIM^2 stitching analysis of an enantiomeric mix of Arg, Asp, Cys, Gln, Ile, Ser, Trp and Val (1 µM each), L-Phe and L-Pro (10 µM each) and Cu^{II} (20 µM) (4 mobility segments: 0.70-0.85, 0.80-0.95, 0.90-1.05 and 1.00-1.15 V.s/cm^2, **Scheme S1**). The ion mobility signals are extracted from the entire acquisition. Note that artifacts signals ("edge effects") appear using this data processing (see **Fig. S7** *versus* **Fig. S8**), and are highlighted at $1/K_0$ values of 0.85, 0.95 and 1.05 V.s/cm^2 (limits of each SIM^2 window range).

(Inserted) Zoom on the extracted ion mobility signals for the complex $[Arg,Pro\text{-}H+Cu^{II}]^+$ showing the mobility shift induced by the artifact signal processing (i.e. extracted from the entire acquisition).

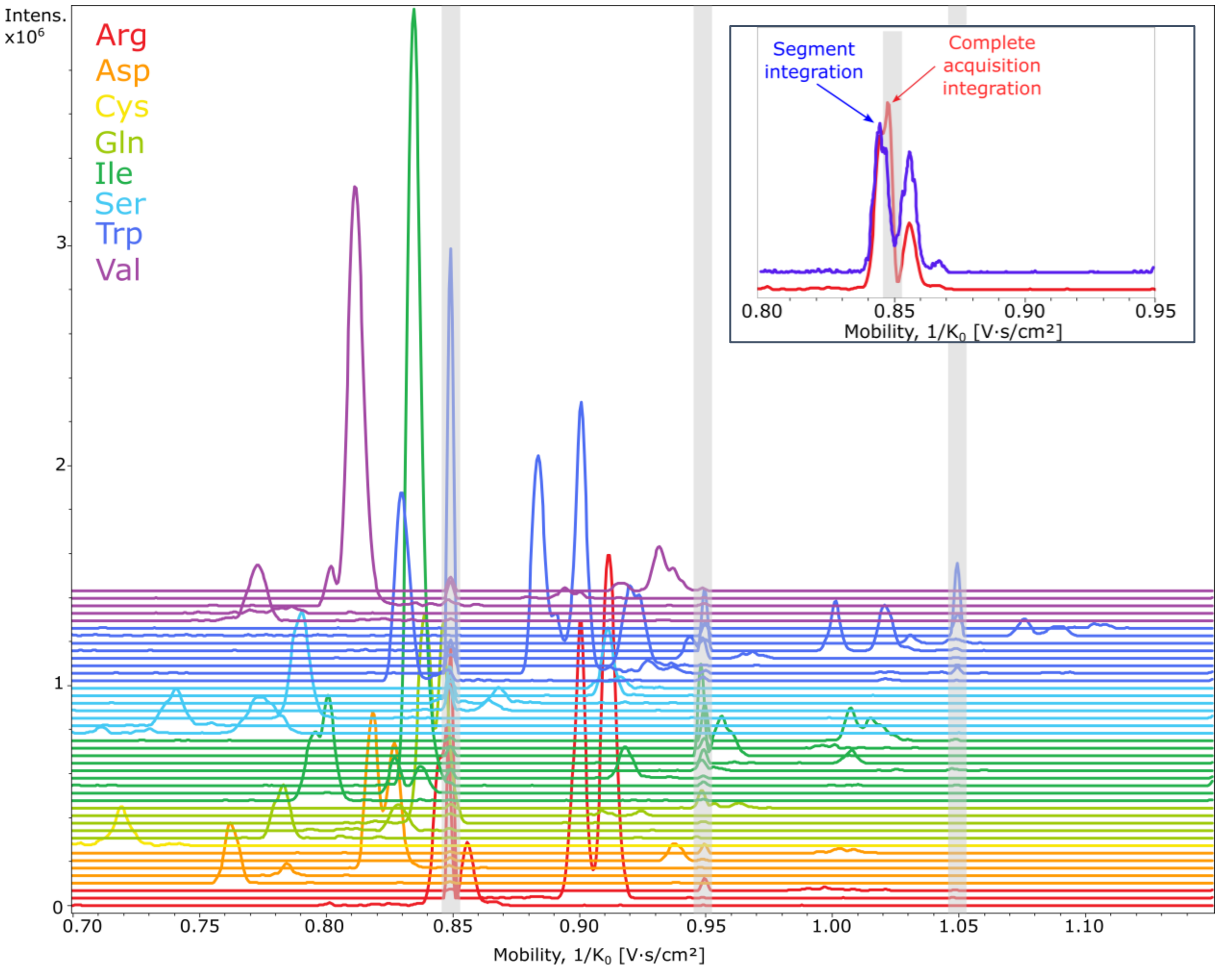

Fig. S9. Extracted ion mobility spectra of the ions detected from the SIM^2 stitching analysis of an enantiomeric mix of Ala, Asn, Glu, His, Leu, Lys, Met, Thr and Tyr (1 µM each), L-Phe and L-Pro (10 µM each) and Cu^{II} (20 µM). Note that the reported ion mobility signals are extracted only from a mobility segment that include each ion of interest (either 0.70-0.85, 0.80-0.95, 0.90-1.05 or 1.00-1.15 V.s/cm^2, **Scheme S1**) in contrast to **Fig. S10** where the ion mobility spectra at given *m/z* ratio are averaged onto the entire acquisition.

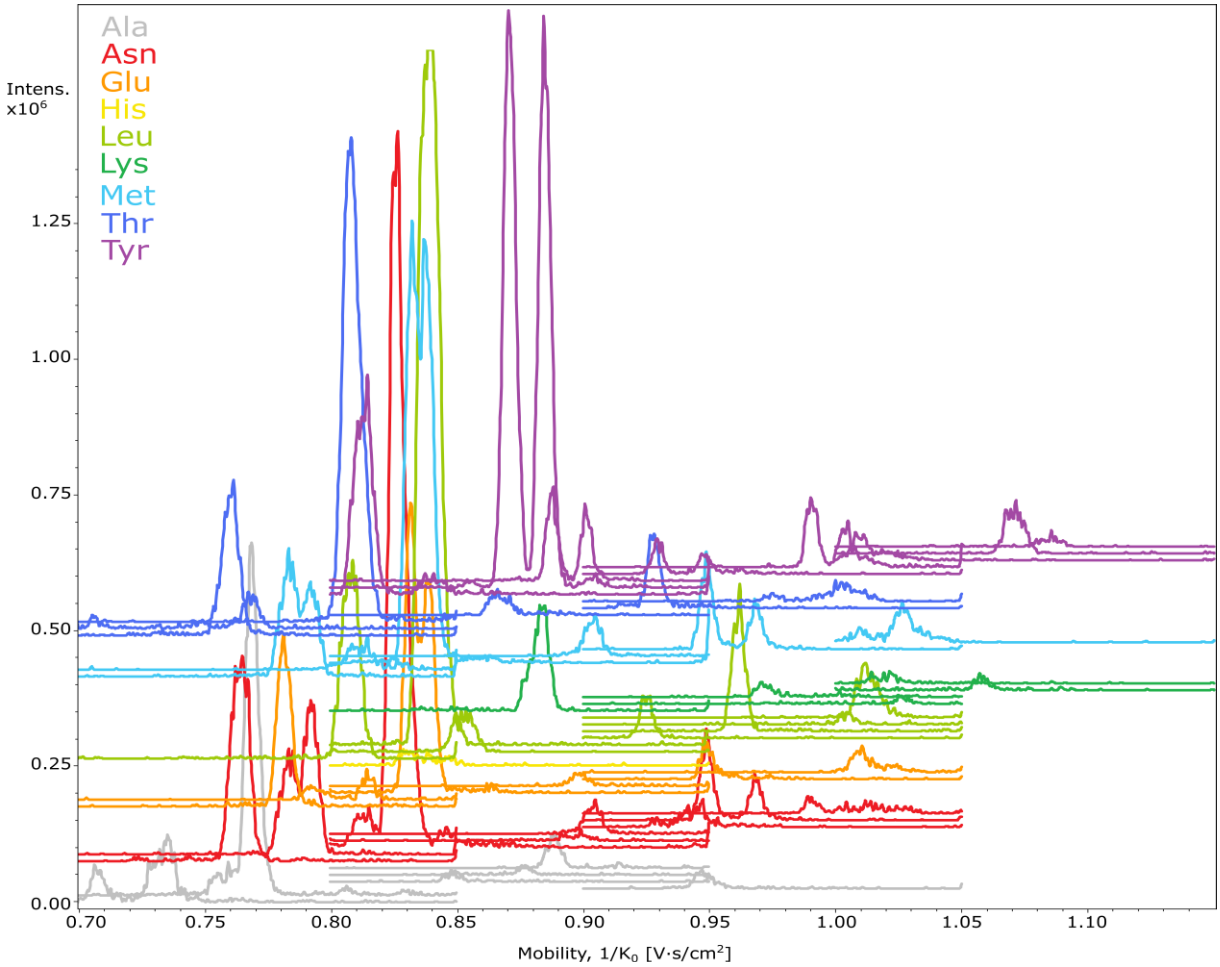

Fig. S10. Extracted ion mobility spectra of the ions detected from the SIM^2 stitching analysis of an enantiomeric mix of Ala, Asn, Glu, His, Leu, Lys, Met, Thr and Tyr (1 µM each), L-Phe and L-Pro (10 µM each) and Cu^{II} (20 µM) (4 mobility segments: 0.70-0.85, 0.80-0.95, 0.90-1.05 and 1.00-1.15 V.s/cm^2, **Scheme S1**). The ion mobility signals are extracted from the entire acquisition. Note that artifacts signals ("edge effects") appear using this data processing (see **Fig. S9** *versus* **Fig. S10**), and are highlighted at $1/K_0$ values of 0.85, 0.95 and 1.05 V.s/cm^2 (limits of each SIM^2 window range).

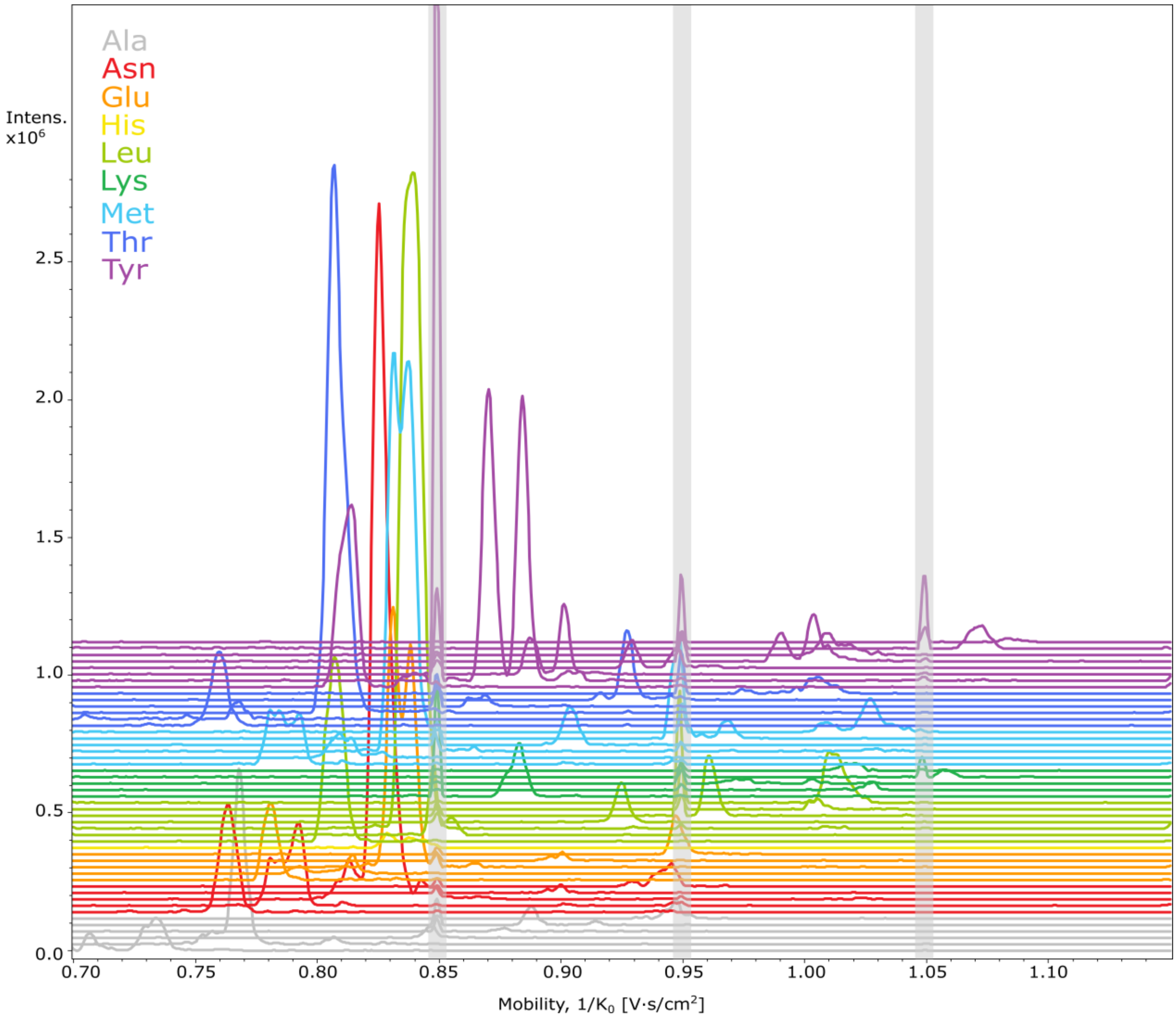

Supplement: Supplementary file 1 — Supplementary file1 (PDF 2701 KB) [file 216_2024_5399_MOESM1_ESM.pdf]
